# Extensive Intra-Kingdom Horizontal Gene Transfer Converging on a Fungal Fructose Transporter Gene

**DOI:** 10.1371/journal.pgen.1003587

**Published:** 2013-06-20

**Authors:** Marco A. Coelho, Carla Gonçalves, José Paulo Sampaio, Paula Gonçalves

**Affiliations:** Centro de Recursos Microbiológicos, Departamento de Ciências da Vida, Faculdade de Ciências e Tecnologia, Universidade Nova de Lisboa, Caparica, Portugal; Duke University Medical Center, United States of America

## Abstract

Comparative genomics revealed in the last decade a scenario of rampant horizontal gene transfer (HGT) among prokaryotes, but for fungi a clearly dominant pattern of vertical inheritance still stands, punctuated however by an increasing number of exceptions. In the present work, we studied the phylogenetic distribution and pattern of inheritance of a fungal gene encoding a fructose transporter (*FSY1*) with unique substrate selectivity. 109 *FSY1* homologues were identified in two sub-phyla of the Ascomycota, in a survey that included 241 available fungal genomes. At least 10 independent inter-species instances of horizontal gene transfer (HGT) involving *FSY1* were identified, supported by strong phylogenetic evidence and synteny analyses. The acquisition of *FSY1* through HGT was sometimes suggestive of xenolog gene displacement, but several cases of pseudoparalogy were also uncovered. Moreover, evidence was found for successive HGT events, possibly including those responsible for transmission of the gene among yeast lineages. These occurrences do not seem to be driven by functional diversification of the Fsy1 proteins because Fsy1 homologues from widely distant lineages, including at least one acquired by HGT, appear to have similar biochemical properties. In summary, retracing the evolutionary path of the *FSY1* gene brought to light an unparalleled number of independent HGT events involving a single fungal gene. We propose that the turbulent evolutionary history of the gene may be linked to the unique biochemical properties of the encoded transporter, whose predictable effect on fitness may be highly variable. In general, our results support the most recent views suggesting that inter-species HGT may have contributed much more substantially to shape fungal genomes than heretofore assumed.

## Author Summary

Genes are commonly vertically inherited, meaning that they share the evolutionary history of the organisms in which they are found. However, they can also be transmitted between species with overlapping niches, a phenomenon known as horizontal gene transfer (HGT) that can occur between closely related species but also between organisms belonging to different domains of life. While HGT is very common in prokaryotes, it has been less frequently reported in eukaryotes, including eukaryotic microbes. In fungi, several instances of genes acquired by HGT from bacteria have been reported, but gene exchange between fungal species is thought to be rare. Here, we describe our findings concerning a single fungal gene that seems to have been transferred between fungi very often. We believe this may be related to the fact that the gene can be both very useful and detrimental for the host, depending on genetic background and environment. Our results suggest that exchange of genes between fungi may happen much more frequently than assumed so far.

## Introduction

Gene gain and loss are deemed to be important mechanisms underlying adaptation to different lifestyles across all domains of life. For many prokaryotes this reflects in the relative sizes of the “core genome”, shared by all individuals of a species, and the so called accessory genome that equips the cells for survival in specific environments and can represent as much as 60% of the total genome [1]. This plasticity is generally thought to be related to the relative ease with which prokaryotes are able to discard genes that are not required, as well as to the diversity and effectiveness of mechanisms mediating gene acquisition [2]. Whereas it is commonly accepted that vertical descent with modification as well as gene duplication followed by divergence of the resulting paralogous genes are paramount for the expansion of gene diversity in prokaryotes, horizontal gene transfer (HGT) between species follows closely in importance, since it seems to have been a very frequent source of genetic novelty throughout the evolution of both Archaea and Bacteria [2], [3]. Many HGT events became evident from the comparison of large numbers of genomes because this allowed the clarification of the phylogenetic relationship between genes that appeared to be paralogous upon examination of a single genome. Indeed, some paralog pairs turned out on more detailed phylogenetic examination to be composed of a “resident” gene and a homolog acquired from a different species, constituting instances of so called “pseudoparalogy” [2]. In other cases, the “resident” gene seemed to have been replaced by a homolog acquired from a different species, an event dubbed “xenolog gene displacement” [2].

Compared with bacteria, nuclear gene content in eukaryotes is generally considered less variable. As possible reasons for this, it has been suggested that meiosis may constitute a limitation to innovation involving large portions of the genome, because it imposes the requirement for pairing between homologous chromosomes [1]. Other reason could be the narrower scope of mechanisms available to eukaryotic cells for the incorporation of DNA from the outside and the necessity that genetic alterations affect the germ line. On the other hand, typical eukaryotic mechanisms like phagocytosis and endosymbiosis seem to have facilitated the acquisition of nuclear genes by protist lineages [4], [5]. As eukaryotic organisms, fungi share some of these limitations but have also distinctive characteristics. Their ability to propagate asexually and the absence of distinction between soma and germ line would be expected to be more permissive with respect to genome changes. In fact, and contrary to earlier assumptions, the large amount of genomic information available for fungi has brought to light important variability within and between closely related species [6], which is often associated to particular chromosomal locations, like subtelomeric regions [7]. Even so, gene repertoires seem to be much more similar among closely related fungal species than observed for prokaryotes [8], [9]. In line with this, HGT is still considered to be generally infrequent, although several well-supported events of acquisition of genes from bacteria [10], [11] and transfers among fungal species have been reported and are contributing to change this view [12]–[15]. While currently available information still falls short of supporting a major role for HGT in the dynamics of fungal genomes, some functional categories of genes stand out as being more prone to be horizontally transferred, as seems to be the case for gene clusters involved in nutrient assimilation [16], the production of secondary metabolites [17], [18] and also for membrane transporters [19], [20]. In fact, evolution of the latter functional class of genes is uncommonly dynamic, as the incidence of duplications and other copy number changing events seems to be particularly high. [21]–[23]. The evolutionary plasticity of transporter genes is possibly linked to the fact that a single gene may have a dramatic (positive) effect on organismal fitness, for example by playing an important role in detoxification processes [24] or by providing a decisive competitive edge in the struggle for nutrients [20], [25], [26]. The latter is particularly crucial for fungi because, unlike many other eukaryotic microbes, they lost the capacity for phagotrophy and thus rely completely on osmotrophic feeding for growth [20].

In the model yeast *Saccharomyces cerevisiae*, evolution of the hexose transporter *HXT* gene family, which belongs to the Major Facilitator Superfamily [27] was studied in detail and a link between expansion of the family and the extant ability of the species to rapidly ferment glucose and fructose was proposed [22]. In this species, hexose transport relies entirely on the *HXT* transporter gene family that also includes a galactose transporter and two glucose sensors [22]. However, two closely related species, *S. uvarum* and *S. eubayanus* (as well as the derived hybrid and domesticated species *S. bayanus* and *S. pastorianus*) possess an additional gene, *FSY1*, encoding a protein specialized in high affinity, specific fructose uptake [28], [29]. Unlike the Hxt proteins, Fsy1 is a proton symporter that accepts fructose and sorbose as substrates but is unable to transport glucose, a highly unusual feature among hexose transporters [30]. The affinity of Fsy1 for fructose is at least one order of magnitude higher than that of the Hxt transporters and may therefore confer substantial advantage in environments with low fructose concentrations [28], [31]. However, Fsy1 was also recently shown to operate under certain circumstances in a mode that co-transports more than one proton with one fructose molecule, which is energetically very costly and unprecedented in fungal hexose transporters [31]. We recently proposed that this departure from the normal 1∶1 stoichiometry might represent a “defective” mode of operation of the Fsy1 transporter, observed only when it functions at high glycolytic fluxes [31]. In two of the species where the *FSY1* gene occurs naturally (*S. bayanus* and *S. pastorianus*), it was shown to be stringently repressed by high fructose concentrations, which normally concur with a high glycolytic flux [32]. Thus, in the presence of abundant fructose, strong repression of the gene prevents Fsy1 from functioning as a fructose driven ATP consuming device with little or no advantage for the cell because fructose transport via the Hxt proteins is effective under these conditions. This constitutes therefore an instance of a single gene that seems to be able to confer on its own appreciable fitness advantages (fructose scavenging capacity) as well as disadvantages (serious deleterious impact on the cell energy metabolism), depending on the environmental conditions and genetic background.

In addition to the gene first isolated from *S. pastorianus* [28], two Fsy1 homologues were functionally characterized in the yeast *Kluyveromyces lactis* [33] and the filamentous fungus *Botryotinia fuckeliana* (*Botrytis cinerea*) [34]. Surprisingly however, the Fsy1 homolog most recently characterized originates from *S. cerevisiae* wine strain EC 1118 and is located within a 17 kb fragment that was horizontally transferred from an unidentified yeast lineage [14], [35]. Efficient fructose metabolism is important in the wine industry because lack thereof is thought to result in stuck or sluggish fermentations with important economic repercussions [36]. However, a clear link between advantageous properties of wine strains and the presence of the *FSY1* gene remains to be established.

These findings concerning an HGT event involving Fsy1, together with the discovery of higher proton∶fructose stoichiometries during Fsy1 operation at high glycolytic fluxes, spurred us to investigate in detail the evolutionary history of the *FSY1* gene, in order to uncover its origin and distribution in fungi and to identify lineage specific losses and putative additional events of HGT. Our hypothesis was that the potentially ambiguous effect of Fsy1 on organismal fitness might affect the pattern of inheritance of the gene. For example, it is conceivable that the gene might be rapidly lost if the organism thrives in environments with high fructose concentrations that can be easily taken up by Hxt-like facilitators without energy expenditure. The fitness disadvantage associated with the presence of the *FSY1* gene under these circumstances would be even more pronounced if stringent transcriptional repression is concomitantly relieved, allowing expression of the gene at high fructose concentrations, when operation of Fsy1 dissipates at least double the amount of ATP [31]. On the other hand, in environmental conditions with low fructose concentrations and scarcity of other carbon sources, the fructose scavenging capabilities of Fsy1 are expected to have a clear positive effect on fitness. In line with this hypothesis, we found a highly dynamic pattern of *FSY1* gene losses and independent acquisitions by HGT, whose frequency is, to the best of our knowledge, unparalleled by any other single gene in fungi.

## Results

### Identification of Fsy1 homologues in fungal genomes

**Figure 1:**
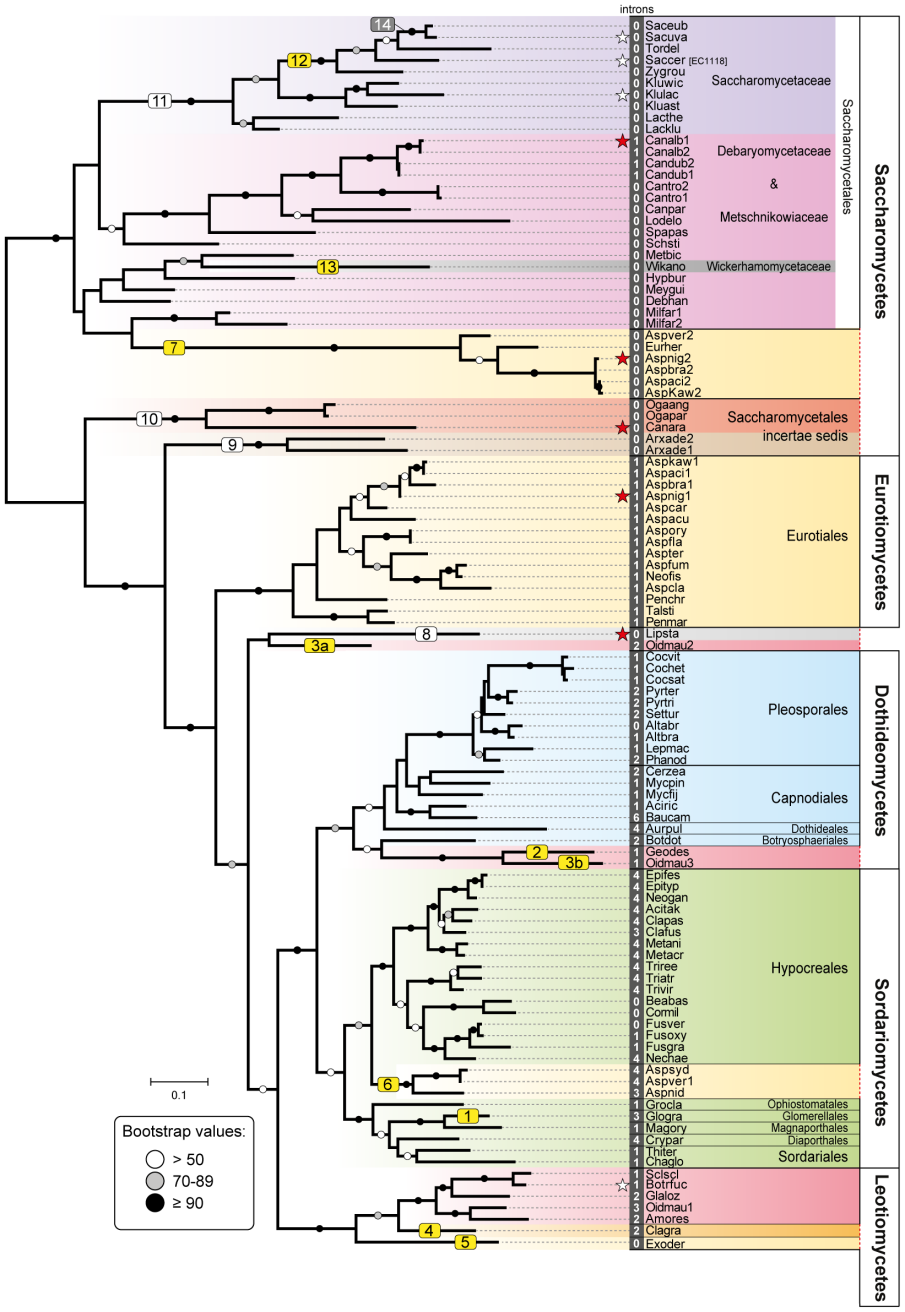
Phylogeny of Fsy1 transporters. Phylogeny based on the amino acid sequence of all Fsy1 homologues identified in the Pezizomycotina and the Saccharomycotina. For species possessing multiple Fsy1 homologues, the numbers ‘1’, ‘2’ or ‘3’ are added to the species designation. Background colors refer to the supra-generic taxa to which each species belongs. White numbers before species names denote the number of introns found in each *FSY1* gene. Putative HGT events discussed in the text are numbered sequentially in boxes: yellow, HGT events supported by topology tests; white, putative HGT events not supported by topology tests; grey, HGT events that cannot be tested. *FSY1* genes functionally characterized in this study or in previous studies are indicated by red and white stars, respectively. The tree is rooted at the midpoint. Bootstrap support values are depicted in tree branches (>50%) as given in the key. Species names are abbreviated as given in Table S1.

**Figure 2:**
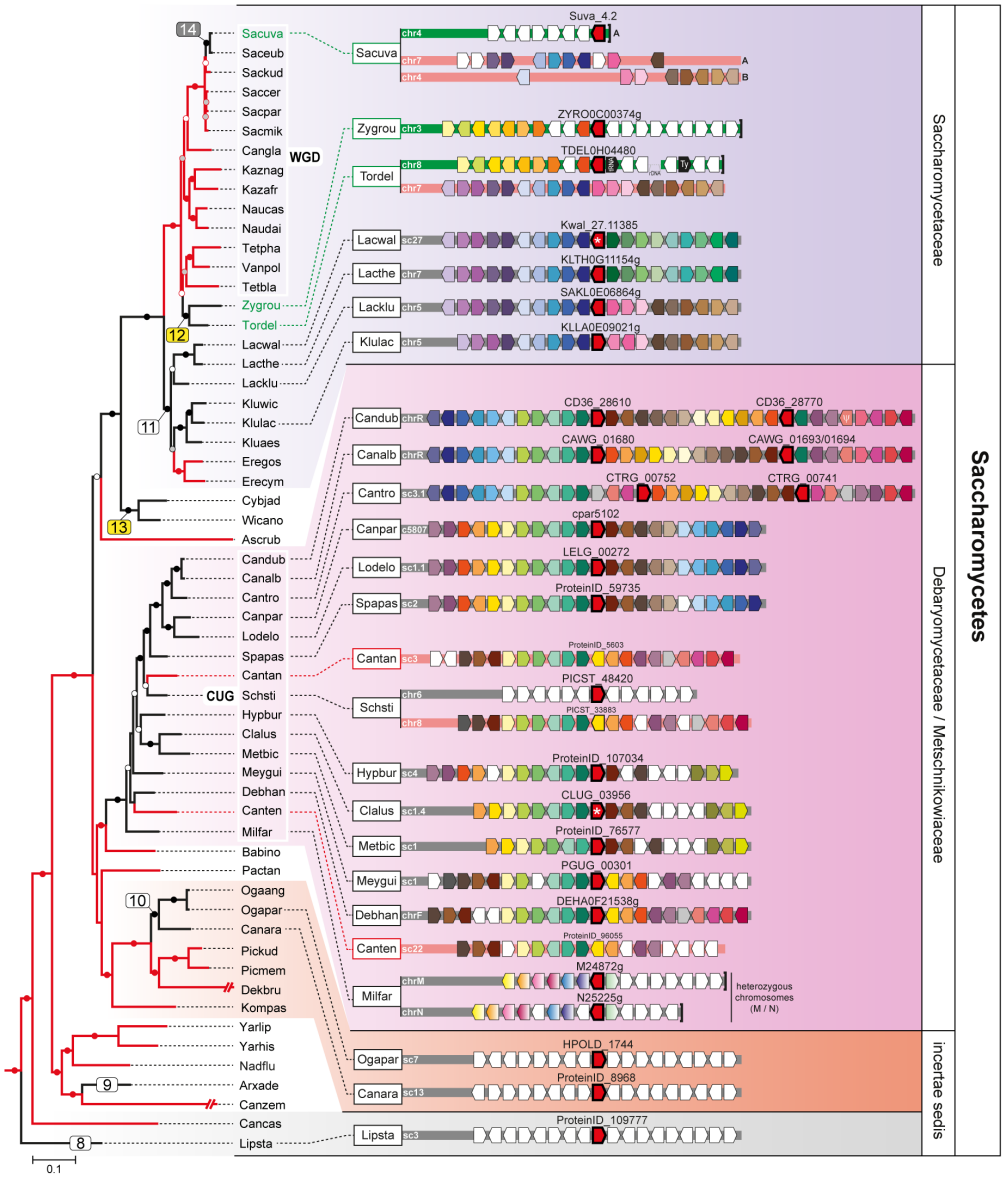
Gene content and organization in *FSY1* loci in Saccharomycetes. Chromosomal regions (chr) or scaffolds (sc) encompassing *FSY1* gene are depicted by grey bars for most of the Saccharomycetes species represented in the species tree (of which a subsection is shown on the left). Pink bars represent syntenic regions where *FSY1* gene is absent (e.g. is *Candida tanzawaensis* and *Candida tenuis*) and those in green indicate instances where *FSY1* gene was likely acquired by HGT. The *FSY1* gene is shown as a strongly outlined red arrow in the center, denoting transcriptional orientation. Within each clade (highlighted by a different background color), orthologous genes are depicted in the same color. Non-syntenic genes are colored white and those in black represent tRNA or Ty elements. The end of a chromosome is indicated by a bracket next to a gene (e.g. see *FSY1* location in *S. uvarum*). Partial *FSY1* gene sequences are indicated with an asterisk. Gene accession numbers are only shown for *FSY1* or immediate flanking genes as they appear in their respective genome databases. The remaining labels are as in Figures 1. Species names are abbreviated as given in Table S1.

**Figure 3:**
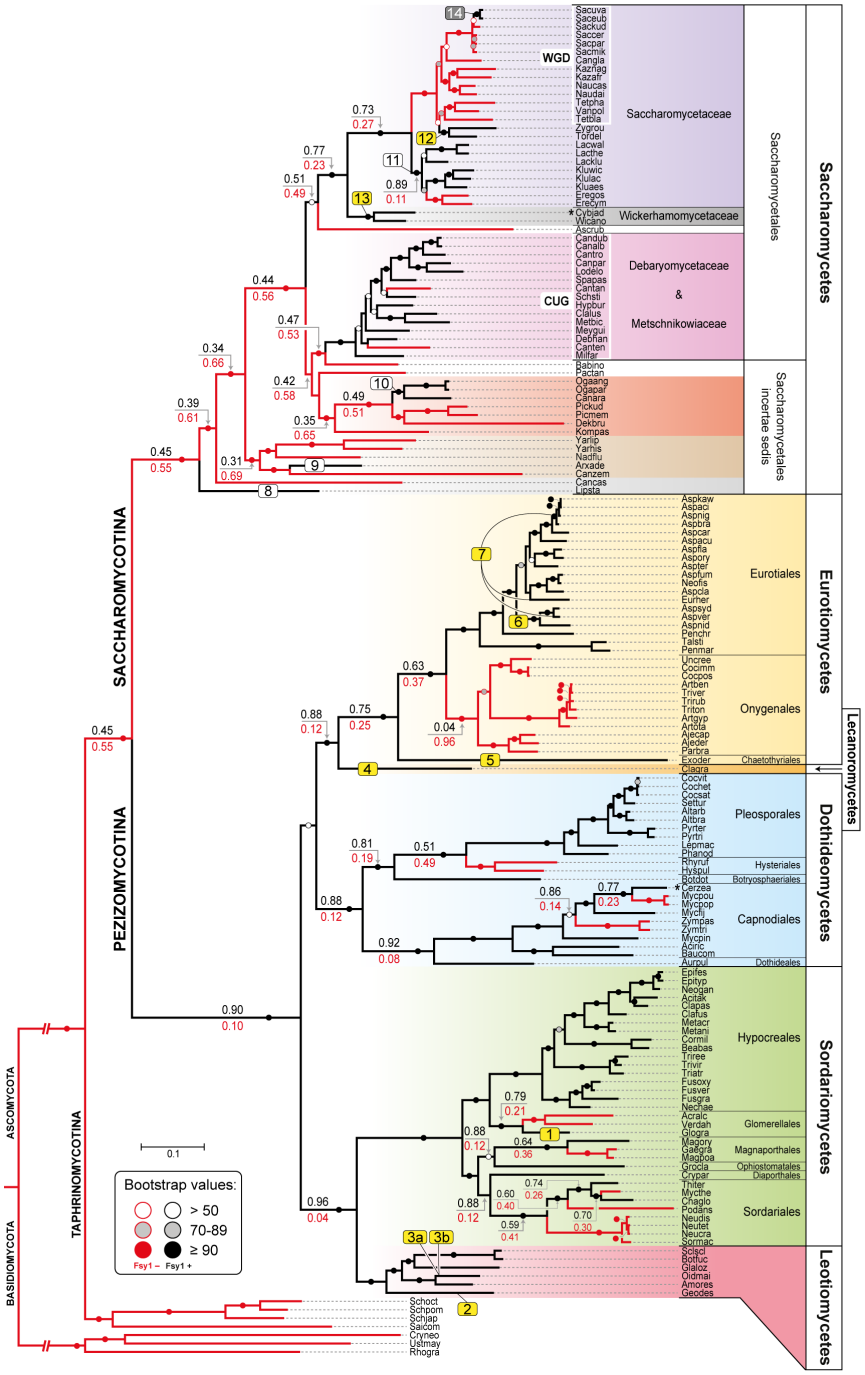
Species phylogeny and *FSY1* ancestral state reconstruction. Phylogeny based on the concatenated amino acid sequences of six RNA polymerase subunits (see methods) from all the species in the Ascomycota surveyed for the presence of Fsy1 and for which whole genome data was available. Four species from the Basidiomycota were used as outgroup. Background colors refer to different supra-generic taxa (Family in the Saccharomycotina and Class in the Pezizomycotina). The label “WGD” indicates species that underwent Whole Genome Duplication. Tree branches depicted in black denote cases where the Fsy1 gene is present in the extant species and red lines indicate branches that lack an Fsy1 gene. Values on tree branches denote the likelihood of Fsy1 being present in the ancestors and are reported as proportional likelihoods, as determined by ancestral state reconstruction. For branches with no values, the likelihood is >0.95 for the presence or absence of Fsy1, as indicated by the color of the lines in the derived branches. Bootstrap support values are depicted in tree branches (>50%) as given in the key. The remaining labels are as in Figure 1. Species names are abbreviated as given in Table S1.

A total of 241 available fungal genomes were surveyed for the presence of Fsy1 homologues using BLAST searches (Table S1). Although it harbors the canonical sugar transporter signature sequences [27], the Fsy1 protein can be clearly distinguished from other fungal sugar transporters, which facilitated the identification of 109 Fsy1 homologs used to construct the phylogenetic tree shown in Figure 1. An *E*-value of 1e-80 was found to be appropriate to distinguish Fsy1 homologs from other transporters, as shown by the comprehensive phylogenetic tree in Figure S1. In most cases, microsynteny conservation in the chromosomal regions surrounding the *FSY1* gene consubstantiated the orthologous relationship between genes of closely related species identified through BLAST searches (Figures 2 and S2). Interestingly, in a phylogenetic tree including all the Fsy1 homologues identified (Figure 1), the proteins encoded by species belonging to the Pezizomycotina (filamentous fungi) and Saccharomycotina (yeasts) do not segregate strictly according to the sub-phyla to which they belong. This prompted us to compare in more detail the topology of the Fsy1 tree with that of the species phylogenetic tree and phylogenetic network depicted in Figure 3 and S3, respectively. The tree and network are based on the concatenated amino acid sequences of six RNA Polymerase subunits previously used in a study with a similar phylogenetic scope [37] and are globally in good agreement with the accepted topology for the different groups within the Ascomycota [38]–[41]. Comparison of the topologies of the trees in Figures 1 and 3 brings to light several clear inconsistencies between the two. These inconsistencies are found among the Saccharomycotina and Pezizomycotina Fsy1 homologs, but most notably among *Aspergillus* species (Figures 1 and 3). Hence, a global analysis of the phylogenies suggests that several Fsy1 homologs have had disparate evolutionary ancestries, which cannot be reconciled with a pattern of transmission of the *FSY1* gene that involves solely vertical inheritance.

### The origin of the *FSY1* gene

*FSY1* seems to be absent in basal fungal lineages (namely Microsporidia, Mucoromycotina, Blastocladiomycota, Chytridiomycota) and in Basidiomycota (see Table S1 for a complete list of the species surveyed). On the contrary, the gene is very common in the Ascomycota with the exception of the earliest derived sub-phylum Taphrinomycotina, where none of the four genomes examined was found to encode a Fsy1 homologue (Figure 3). In the remaining sub-phyla, Pezizomycotina and Saccharomycotina, *FSY1* distribution is patchy, punctuated by losses common to entire lineages (e.g. the entire order Onygenales) or limited to a few species within an otherwise Fsy1 harboring clade (e.g. the order Capnodiales). Using the species tree depicted in Figure 3, we employed an ancestral state reconstruction method to estimate the likelihood of *FSY1* being present in ancestors of extant lineages represented by the various internal nodes in the tree. This analysis suggests that the gene may have originated in the Pezizomycotina, being later horizontally transferred to the Saccharomycotina after the divergence of several basal yeast lineages (Figure 3).

### Evolution of Fsy1 in the Pezizomycotina

Nearly half of the Pezizomycotina species lacking a *FSY1* gene belong to the order Onygenales that includes dimorphic human pathogenic fungi such as *Paracoccidioides brasiliensis* [42]. In fact, none of the genomes from species in the Onygenales was found to encode Fsy1 homologues, while in the sister clade (Eurotiales) the reverse situation is observed and the most recent common ancestor (MRCA) of both clades is predicted to have possessed the gene (Figure 3). The MRCAs of the orders Hysteriales and Glomerellales also seem to have lost the gene, although in these cases only a few species were examined in each order. All the remaining orders exhibit at least one *FSY1* gene loss with the exception of the Hypocreales and the Pleosporales in which all the genomes examined encode a Fsy1 homologue (Figure 3).

The phylogeny of Pezizomycotina Fsy1 homologues shows in addition that some species are devoid of a cognate *FSY1* gene but acquired a *FSY1* gene from another lineage, seemingly by HGT. In addition to several cases in the Eurotiales described in detail in the next section, this was observed for two other species. As shown by the species and Fsy1 phylogenies in Figure 1 and Figure 3, the *FSY1* gene from *Glomerella graminicola* clusters within the Magnaporthales (event 1), while *Geomyces destructans* (Leotiomycetes) obtained its copy from a lineage related to the Botryosphaeriales (Dothideomycetes) (event 2, Figures 1 and 3). Both events are supported by topology comparison, using the Shimodaira-Hasegawa (SH) test, in which these *FSY1* genes are constrained to occupy their expected place in the phylogeny (*P*<0.01; Figure S4 and Table S2). In addition, loss of synteny is observed in the regions surrounding the *FSY1* gene in *G. destructans* (Figure S2), which is in line with its acquisition via HGT.

The mycorrhizal fungus *Oidiodendron maius* is the only species examined that exhibits three *FSY1* genes (Figures 1 and 3, events 3a and 3b). This species not only retained its cognate *FSY1* copy but also acquired two additional genes by HGT in events that are supported both by topology testes (*P*<0.01; Figure S4 and Table S2) and by synteny analysis (Figure S2). One xenolog is phylogenetically nested in the Pezizomycotina but it is not possible to identify unequivocally a donor lineage (Figure 1 and Figure 3, event 3a). The second clusters with high support with the *FSY1* gene from *G. destructans* also acquired by HGT (see above and Figure 1, event 3b). Both species belong to the Leotiomycetes, but the remaining species examined in this clade (*Amorphotheca resinae*, *Botryotina fuckeliana*, *Sclerotinia sclerotiorum* and *Glarea lozoyensis*), which are more closely related to *O. maius* than *G. destructans*, lack a similar gene acquired by HGT. This could mean that the MRCA of these species was the recipient of the transferred gene that was subsequently lost after the divergence of *G. destructans* from the other lineages.

The Fsy1 homologues found in *Cladonia grayi* and *Exophiala dermatitidis* cluster together with the Leotiomycetes with high bootstrap values (Figures 1 and S5), but the two species belong to the Eurotiomycetes and Lecanoromycetes, respectively (Figures 3 and S3). These seem to constitute yet two independent HGT events (numbered 4 and 5, Figures 1 and 3), which are supported by topology tests (*P*<0.01; Figure S4 and Table S2), but insufficient sampling prevents identification of a donor lineage.

Finally, we identified a very recent inactivation of the *FSY1* gene in *Cercospora zeae-maydis* (Capnodiales) caused by a transposon insertion within the coding region. This event seems to be very recent because the gene displays no sign of degradation and the phylogenetic position of the *in silico* translated protein is in line with the species phylogeny (Figures 1 and 3).

### Three distinct origins for Fsy1 homologs in *Aspergillus*

**Figure 4:**
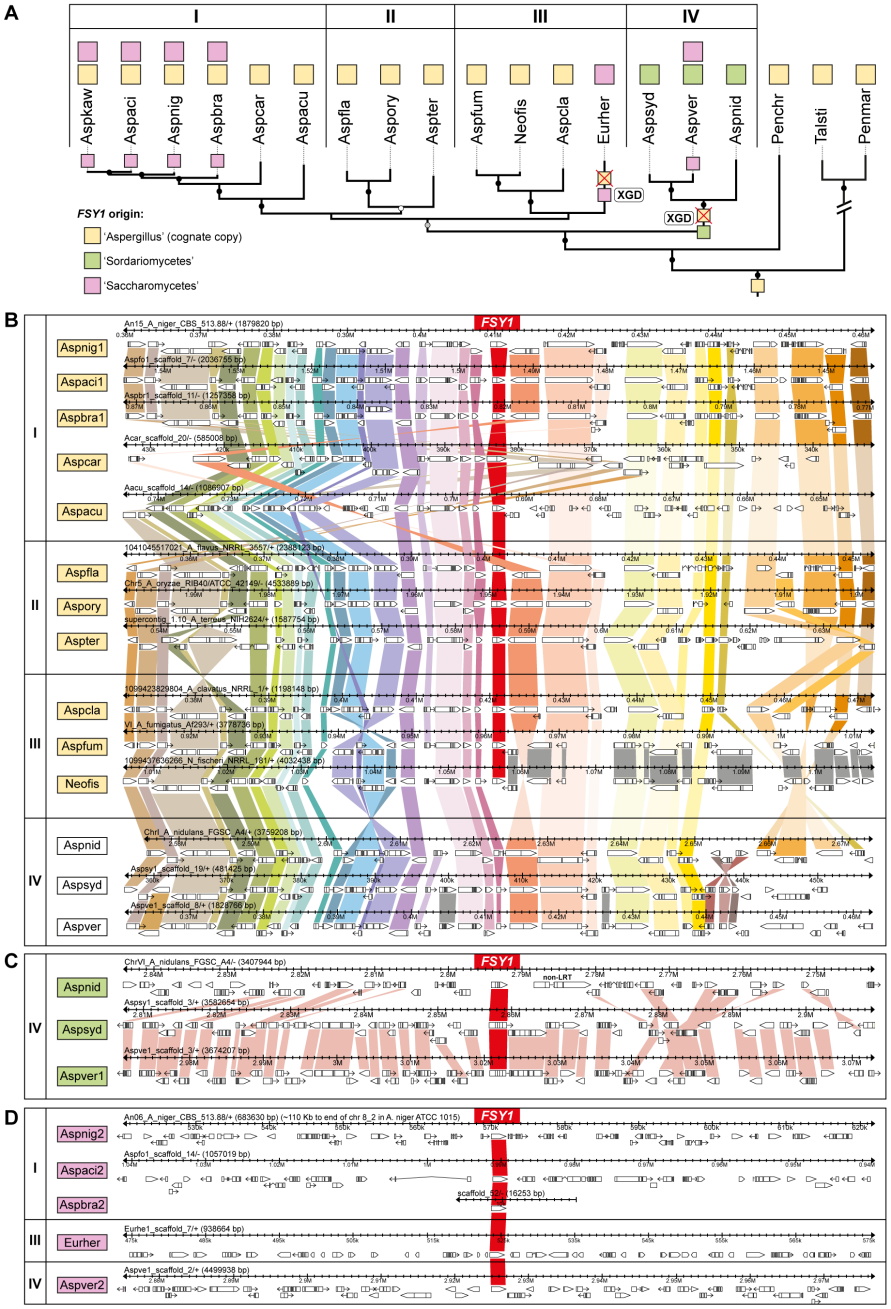
Gene content and organization in *FSY1* genomic region in the Eurotiales. (A) Subsection of the tree depicted in Figure 3 underlining possible events leading to the extant distribution of *FSY1* genes in species of Eurotiales. The three types of *FSY1* genes are depicted as squares and colored according to their most likely origin as shown in the key. The most likely point of acquisition of each of the genes is indicated by a colored square on the respective tree branch. *FSY1* gene losses (possibly due to xenolog gene displacement – XGD) are indicated by red crosses. ‘I’, ‘II’, ‘III’ and ‘IV’ represent different lineages within the Eurotiales. (B) Comparative organization of the cognate *FSY1* locus in Aspergilli species represented in the tree shown in A. Genes are shown as white arrows denoting the direction of transcription and orthologs are connected with vertical bars of the same color. *FSY1* orthologs in the center are connected by red bars. (C) Comparative organization of the genomic region encompassing the *FSY1* gene presumably acquired from the Sordariomycetes by HGT (event 14 in Figure 1) to the MRCA of lineage ‘IV’. In (D) the same is shown for *FSY1* genes acquired by HGT from the Saccharomycetes clade. Only syntenic genes (orthologs) are connected by vertical bars. Species names are abbreviated as in Table S1.

In the order Eurotiales, four species do not encode a cognate Fsy1 (*Aspergillus nidulans*, *Aspergillus sydowii*, *Aspergillus versicolor* and *Eurotium herbariorum*). Two independent *FSY1* gene loss events seem to account for this (Figure 4A). Interestingly, all these species were capable of capturing *FSY1* genes new to the lineage (events 6 and 7 in Figures 1 and 2; Figure 4). To ascertain the nature of this phenomenon, we first asked whether these species might have “accidentally” lost their cognate *FSY1* genes as part of gross deletions or chromosomal rearrangements. To assess this, we examined in detail the chromosomal region where, according to the very well conserved microsynteny among species in the Eurotiales, the cognate *FSY1* gene should have been located in the three Aspergilli that lack a cognate *FSY1* (Figures 4B and S6). Surprisingly, in *A. nidulans, A. versicolor and A. sydowii* the chromosomal region in question retains almost perfect synteny with the closest *Aspergillus* species in the immediate vicinity of the *FSY1* gene with the exception of the absence of the latter and the inversion of a flanking gene (*NEO1*, Figures 4B and S6). In *E. herbariorum* a similar situation is found, but in this case, three genes novel to the region are located at the position occupied by *FSY1* in the species that possess a cognate gene (result not shown). Interestingly, and in addition to these events where apparently only *FSY1* was lost from this region, a number of rearrangements in *Aspergillus carbonarius*, *Aspergillus aculeatus* and *Aspergillus fumigatus* cause an abrupt loss of synteny in the regions immediately downstream of the *FSY1* gene (Figures 4 and S6).

The two types of xenologs found in these four species (*A. nidulans*, *A. sydowii*, *A. versicolor* and *E. herbariorum*) belong to two unrelated phylogenetic groups. The first group encompasses genes that cluster within the Sordariomycetes (order Hypocreales; Figure 1) and were probably acquired from this lineage by the MRCA of *A. nidulans*, *A. versicolor* and *A. sydowii* (event 6, Figures 1 and 3), in a single event that is supported by a topology test that rejects the monophyly of Aspergilli *FSY1* genes (*P*<0.01; Figure S4 and Table S2). In accordance to this hypothesis, the genes exhibit a number of introns (3 or 4 introns; Figure 1) which is more in line with the number of introns found in *FSY1* genes from the presumed donor lineage than with the number of introns found in the cognate *Aspergillus FSY1* genes (1 intron; Figure 1). Inspection of the genomic region in the vicinity of these xenologs in the three species shows an extent of synteny conservation consentaneous with the phylogenetic relation between the species, supporting that the three genes originate from a single HGT event (Figure 4C). The second group of xenologs are found in six species (*Aspergillus kawachii*) and are also closely related to each other (Figure 1, event 7 and Figure 4A). Notably, these genes are phylogenetically closer to the Saccharomycotina Fsy1 homologs, suggesting that they were acquired from an undetermined lineage in this sub-phylum. This putative HGT event (event 7, Figure 1) is also supported both by the absence of introns in their *FSY1* genes and by the SH test that rejected the alternative hypothesis of monophyly of Aspergilli *FSY1* genes (*P*<0.01; Figure S4 and Table S2). Intriguingly, *A. versicolor* possesses two xenologs, one of each of the two groups (Figure 4) although it lacks a cognate *FSY1* gene. Two lines of evidence suggest that the acquisition of the “yeast-like” xenolog by the six species required multiple lateral transfer events. Firstly, given the phylogenetic position of the species that possess this type of xenolog, a single acquisition would have to have occurred in the MRCA of all *Aspergillus* species studied and consequently a large number of independent losses would have to be postulated to explain the present distribution. Secondly, no synteny is found in the vicinity of the “yeast-like” *FSY1* xenolog, even between sibling species like *A. niger*, *A. acidus* and *A. brasiliensis* (Figure 4D). This could mean that the gene underwent multiple (sequential) events of lateral transfer between these species, after it was originally acquired by one of them. However, it should be noted that the “yeast-like” Fsy1 phylogeny is compliant with species phylogeny, raising the possibility that the lack of synteny could be due to the gene being located in a region where synteny is usually absent, like subtelomeric regions. This could hold at least for *A. niger*, where *FSY1* is located at the subtelomere, but not for its close relative *A. acidus* since the *FSY1* gene is located far from the end of the chromosome in this species. In *A. versicolor*, the “yeast-like” *FSY1* is also found in a non-telomeric region that shares synteny with its close relative *A. sydowii*, except for the presence of the *FSY1* gene (Figure S7). This provides additional evidence that the acquisition of the “yeast-like” *FSY1* gene occurred only after speciation. The alternative hypothesis would require two independent events of loss of *FSY1* (in *A. nidulans* and *A. sydowii*) to explain extant *FSY1* distribution.

### Additional lateral transfer events involving Fsy1 in the Saccharomycotina

The first notable feature concerning Saccharomycotina Fsy1 homologs, is the fact that the few examples found in early diverging yeast lineages cluster much more closely than expected to the Pezizomycotina Fsy1 clade, or are even included in that clade. The latter is the case for the Fsy1 homolog from *Lipomyces starkeyi*, the most basal yeast lineage included in this study (event 8, Figures 1 and 3). The homologs found in *Arxula adeninivora* (event 9) and the cluster formed by the Fsy1 proteins found in *Candida arabinofermentans* and in two *Ogatea* species (event 10), seem also to be notably distinct from the remaining Saccharomycotina Fsy1 homologs, since their association with one of the groups defined by either of the two sub-phyla is not well-established (Figure 1). In addition, no synteny is observed in the vicinity of *FSY1* in closely related species of this group (*viz. O. angusta* and *C. arabinofermentans*), contrary to what is seen for the large majority of closely related species carrying a *FSY1* gene (Figure 2). Together, these observations could suggest that the above-mentioned species acquired a *FSY1* gene from the Pezizomycotina by at least three independent HGT events. However, the result of a topology test that compares the topology in Figure 1 with a topology in which the three lineages (*L. starkeyi*, *A. adeninivora* and *C. arabinofermentans*/*O. angusta* and *O. parapolymorpha*) were constrained to occupy their expected places in the Saccharomycotina phylogeny does not support this hypothesis. Likelihood scores associated with these two alternative topologies were not found to be significantly different using the SH test (Figure S4 and Table S2). Hence, using currently available data, we cannot exclude the possibility that *FSY1* was present in the MRCA of the Pezizomycotina and Saccharomycotina. However, it is worth noting that this possibility contradicts the ancestral state reconstruction results and would require the assumption of at least five independent *FSY1* gene losses in basal Saccharomycotina lineages in order to explain extant distribution of the gene. Should on the contrary events 1, 2 and 3 be the result of HGT, than it remains difficult to state where in the evolutionary history of the Saccharomycotina the *FSY1* gene would have been acquired. This is mainly because there is some uncertainty concerning the phylogenetic position of the *C. arabinofermentans*/*Ogatea* lineage (Figure 3 and [43]), which is important to answer this question. In summary, we believe it is premature to conclude firmly about the origin of Sacharomycotina *FSY1* gene and on whether or not the *FSY1* genes in basal lineages have had their origin in HGT events. Further genome sampling in the more basal part of the Saccharomycotina lineage will be required to amend these results.

The remaining Saccharomycotina Fsy1 homologs cluster together (Figures 1 and S5), indicating that they share a common ancestor, the majority of the genes being found in the so-called CUG clade. The CUG clade is a monophyletic clade within the Saccharomycotina in which all the species translate the CUG codon as serine rather than leucine [44]–[46] (Debaryomycetaceae/Metschnikowiaceae; Figures 1 and 3). In this lineage, the genomes of most species encode a Fsy1 homolog and the Fsy1 phylogeny within the clade is generally in accordance with the species phylogeny. In line with vertical transmission of the *FSY1* gene during evolution of the species in the CUG clade is also the observation of conserved synteny in the region surrounding the *FSY1* gene in all species, with the exceptions of *Scheffersomyces stipitis* and of the earliest derived species *Millerozyma farinosa* (Figure 2). However, in the Fsy1 phylogenetic tree (Figure 1), the CUG clade is not monophyletic. This entails that the lineage harbouring *Candida albicans* seems to be more closely related to the clade formed by the Saccharomycetaceae Fsy1 homologs than warranted by the phylogenetic relationship between these taxa. This could also suggest that the evolutionary trajectory of the *FSY1* gene in the Saccharomycetaceae was initiated by an event of HGT from the *C. albicans* lineage to the MRCA of the *Kluyveromyces/Lachancea* lineage. However, this putative occurrence (event 11, Figures 1 and 3) is not supported by topology tests where the CUG clade is forced to be monophyletic (Figure S4 and Table S2). Finally, according to the species phylogeny, the Fsy1 homologs from *Kluyveromyces* and *Lachancea* should be more closely related to each other than to the Fsy1 homologues from *Zygosaccharomyces* and *Torulaspora*, but the phylogeny supports the opposite (Figures 1 and 3). This could imply that the gene was horizontally acquired by the MRCA of *Zygosaccharomyces* and *Torulaspora* from the *Kluyveromyces* lineage (event 12, Figures 1 and 3). In fact, while the region where the *FSY1* gene is normally located in the *Kluyveromyces*/*Lachancea* lineage exhibits very strong synteny between the latter lineage and *Torulaspora delbrueckii*, *FSY1* is absent from that region in *T. delbrueckii* and appears rather at a subtelomeric region exhibiting synteny between *T. delbrueckii* and *Z. rouxii* (Figure 2). These observations are in line with the cognate *FSY1* gene being lost in the MRCA of the *Torulaspora/Zygosaccharomyces* lineage and with the acquisition by this lineage of a novel gene from the *Kluyveromyces* branch. The result of a topology test constraining the *Kluyveromyces* and *Lachancea* Fsy1 homologs to conform to the species phylogeny contradicts the null hypothesis of vertical inheritance (*P*<0.05; Figure S4 and Table S2) and thus supports the occurrence of this HGT event.

Another clear conflict between Fsy1 phylogeny and the species phylogeny concerns the Fsy1 homologs from *Cyberlindnera jadinii* and *Wickerhamomyces anomalus* (event 13, Figures 1 and 3). Of these two species, only the latter exhibits a putatively functional *FSY1* gene because in the former a heavily degenerated pseudogene is found. The *W. anomalus* Fsy1 homologue is phylogenetically nested within the *Debaryomyces* branch of the CUG clade (Figure 1) while the established species phylogeny places this species in a basal position relatively to the Saccharomycetaceae clade (Figures 3 and S3). Therefore, this species, or possibly the MRCA of *W. anomalus* and *C. jadinii* presumably also acquired *FSY1* by HGT from a donor in the *Debaryomyces* lineage (event 13, Figures 1 and 3). This putative HGT event is also supported by topology tests (*P*<0.05; Figure S4 and Table S2).

The most important instance of *FSY1* gene loss involving a large number of species, is observed in the clade that comprises all the yeasts descending from a common ancestor that underwent a Whole Genome Duplication (WGD) approximately 100 million years ago [47]. The pattern of distribution of Fsy1 is in line with the generally accepted view that the *Zygosaccharomyces* lineage is the closest to the WGD ancestor, and with a loss of the *FSY1* gene in the WGD ancestor, since apparently none of the post-WGD genomes encodes cognate Fsy1 homologues (Figures 1, 2 and 3). The two genes in *S. uvarum* and *S. eubayanus* are, together with the gene found in *S. cerevisiae* strain EC1118, the sole instances of genomes encoding Fsy1 in post-WGD yeasts (Figures 1 and 3). The gene in the *S. cerevisiae* strain was formerly shown to have been acquired by HGT [14], [35]. Since the WGD ancestor is predicted with high likelihood to have lost its *FSY1* gene, it seems probable that the common ancestor of the very closely related *S. uvarum* and *S. eubayanus* species also acquired a *FSY1* gene by HGT (event 14, Figures 1 and 3). The phylogenetic position of the *S. uvarum* and *S. eubayanus* genes does not contradict the species phylogeny in this case, which makes it impossible to find support for this event using topology tests. If a HGT event took place, the donor lineage must have been very closely related to the last pre-WGD species, *Z. rouxii* and *T. delbrueckii*. The alternative explanation would be that the *FSY1* gene was present in the WGD ancestor and was lost independently in all the post-WGD lineages except the *Saccharomyces* lineage, which does not seem to be parsimonious and is in contradiction with the predictions of the ancestral state reconstruction analysis (Figure 3).

In addition to multiple events of HGT and the loss of *FSY1* in the WGD ancestor, several independent *FSY1* gene losses have to be postulated since approximately half of the Saccharomycotina species examined do not carry the gene. This is the case for the genus *Eremothecium* (Figure 3), in addition to two independent losses that, in the present limited sample, involve only a single species each (in *Candida tenuis* and *Candida tanzawaensis*, Figure 3). Taking both the phylogeny and the synteny analysis into account (Figure 2) it seems likely that loss of the *FSY1* gene in *C. tanzawaensis* was preceded by a translocation of the gene to a different chromosomal location in the MRCA of *Scheffersomyces stipitis* and *C. tanzawaensis*. This is suggested by the loss of synteny in the region surrounding the *FSY1* gene in *S. stipitis*, a situation unique among the CUG clade species. Finally, if basal Saccharomycotina lineages acquired the *FSY1* gene by vertical inheritance, which, as mentioned above, cannot be excluded in face of available data, at least five additional independent losses have to be postulated in the this region of the Saccharomycotina tree.

We noted that five Saccharomycotina species harbour two *FSY1* genes. Three of these species (*C. albicans*, *C. dubliniensis* and *C. tropicalis*) represent apparently three independent segmental duplication events that took place after speciation, since the paralog pairs encode (nearly) identical Fsy1 proteins. A fourth CUG clade species, *Millerozyma farinosa*, testifies to a different situation since the sequenced strain (CBS 7064) is in fact an interspecies hybrid in the process of resolution [48]. In this case, the two distinct *FSY1* genes are located in two heterozygous chromosomes, one of which was acquired from a different, but closely related species [48]. An older duplication seems to have given rise to the two *FSY1* genes presently found in *A. adeninivorans*.

### Function and biochemical properties of selected *FSY1* homologues

**Figure 5:**
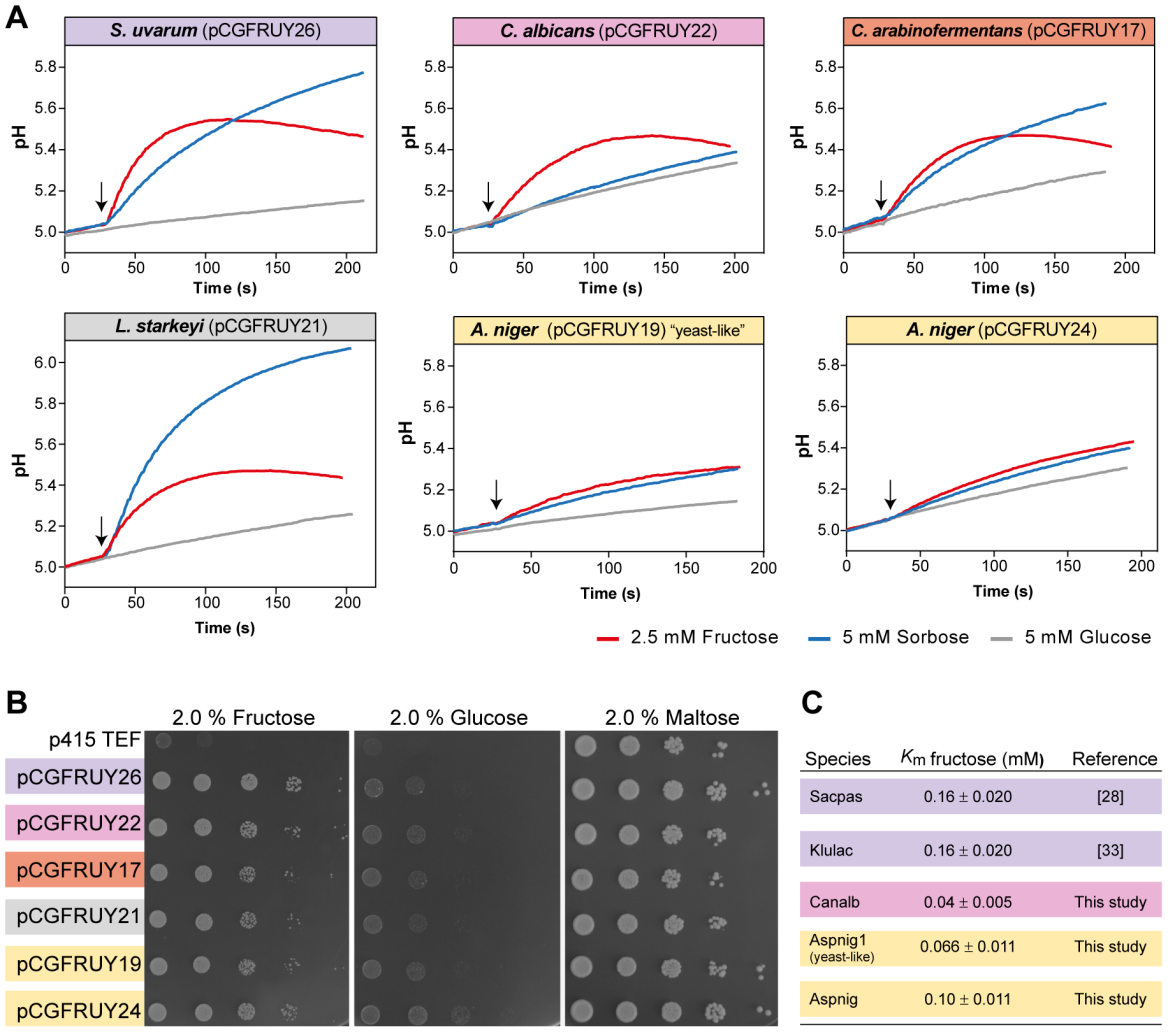
Conservation of Fsy1 function across a broad phylogenetic range. Biochemical properties of Fsy1 homologs of various origins were expressed as sole hexose transporter in a *S. cerevisiae hxt*-null. (A) Fsy1 activity assessed by measurement of the alkalinisation elicited by the addition (marked by the arrows) of fructose (red) or sorbose (blue) to unbuffered cell suspensions of the *hxt*-null strain expressing various Fsy1 homologs, as indicated. Glucose (grey) fails to elicit a pH change in all cases. (B) Growth of the *hxt*-null strain expressing Fsy1 homologs on medium containing fructose, glucose or maltose as sole carbon and energy source, as indicated. (C) Estimated *K*_m_ values for D-[U-^14^C]fructose uptake mediated by various Fsy1 homologs, including those reported in previous studies. Species names are abbreviated as in Table S1. *S. pastorianus* (Sacpas) is a hybrid species harbouring the *FSY1* gene from parental species *S. eubayanus* (Saceub).

The particularly dynamic evolutionary history of *FSY1* raises the question of whether the Fsy1 homologue family may contain proteins whose functional properties underwent substantial changes in one or more lineages in the course of evolution. The best-characterized Fsy1 homologue originally cloned from *Saccharomyces pastorianus* was found to mediate solely the uptake of fructose and sorbose [28], [49]. Fsy1-mediated glucose transport was undetectable in *in vitro* assays and was insufficient to support significant growth on glucose of a *S. cerevisiae* strain devoid of its cognate Hxt transporters [32], [50]. The other Fsy1 homologues characterized so far exhibit similar properties [33], [34]. To evaluate the extent of conservation of Fsy1 function in other phylogenetic lineages, we similarly expressed some of the newly identified genes as sole hexose transporter in *S. cerevisiae*. The Fsy1 homologues tested, indicated in Figure 1, were all capable of complementing growth of the *hxt*-null strain on fructose (Figure 5). In addition, they all appear to operate as H^+^ symporters, since substrate addition to aqueous cell suspensions of strains expressing the various Fsy1 proteins resulted in all cases in transient alkalinisation of the extracellular medium. Moreover, the results shown in Figure 5 suggest that all of the Fsy1 homologues tested accept sorbose as a substrate in addition to fructose, while none is capable of transporting significant amounts of glucose. Notably, Fsy1- mediated sorbose uptake seems to be particularly vigorous in *L. starkeyi*, which contrary to *C. arabinofermentans*, *K. lactis* and *S. uvarum*, is capable of growing on sorbose as sole carbon and energy source. Discrepancies in the strength of H^+^ symport signals observed between the strains expressing the different transporters may simply result from distinct efficiencies in heterologous expression/membrane localization. Therefore, these discrepancies are not informative in what concerns possible functional differences between the transporters when operating in their natural context. In summary, we conclude that Fsy1 function seems to have remained remarkably constant in the course of evolution, possibly with some species-specific adjustments that can be traced back to physiological characteristics of the species to which a particular protein belongs, but apparently without major shifts in substrate preference or mode of operation. This is particularly important to note for the “yeast-like” *A. niger* Fsy1 and the homologs from *L. starkeyi* and *C. arabinofermentans*, all of which are located on long branches of the Fsy1 tree (Figure 1).

## Discussion

Our survey among available fungal genomes brought to light a patchy distribution of *FSY1*, a gene encoding a specific high affinity fructose/H^+^ symporter, in two sub-phyla of the Ascomycota, Pezizomycotina and Saccharomycotina. Two instances of Fsy1 loss in deeper nodes of the phylogeny are very possibly related to important turning-points in lifestyles and genomic make-up of the lineages involved. The first, concerning the Onygenales, is most probably a consequence of a shift from a nutritional association with plants to animals [42], [51] and the reduction of gene families associated with the metabolism of plant material [51]. It seems plausible that *FSY1* is also among the genes that became dispensable in this context. The second instance concerns the ancestor of the *Saccharomyces* lineage that underwent whole genome duplication (WGD). In this case, a switch to a fermentative lifestyle occurred and the number of hexose transporters increased substantially [22]. Our phylogenies suggest that Fsy1 became dispensable and was lost very soon after the WGD, before the first extant post-WGD lineages diverged, possibly as a result of the WGD itself. In addition, several independent and more recent losses in various lineages were noted, but as far as can be judged from available data (for *S. uvarum*, *S. eubayanus* and *A. niger*), there is no intra-species variation in the presence of the *FSY1* gene. Notably, in these three species, as well as in *Z. rouxi* and *T. delbrueckii*, the gene is found in subtelomeric regions (Figures 3 and 4), a genomic location characterized by frequent chromosomal rearrangements and enrichment in transposable elements [52]–[54]. Interestingly, these regions are also known for facilitating the accumulation of genes that are associated with niche-specific adaptations. For example, in *S. cerevisiae* several genes involved in sugar utilization, as well as the *FLO* gene family involved in the flocculation process important in brewing, are located in subtelomeric regions [55], [56].

The identification of instances of horizontal gene transfer is often controversial, mainly because assumptions on their occurrence have been made based on insufficient evidence in the past and the transfer mechanisms are poorly understood [57]. However, sufficient consensus exists that substantial and well-supported incongruences between a gene tree and the accepted species phylogenetic tree is a strong indication of HGT [20], [58]. Likewise, other types of “character-state discordance”, such as patchy phylogenetic distribution of the genetic element along various lineages and inconsistency in sequence patterns between the gene and the resident genome (e.g. number of introns) are also good evidence to support HGT [59].

In the present study, we detected by phylogenetic analyses at least 10 novel and independent events of HGT involving the same gene, *FSY1*. Most of the well-supported HGT involving fungal donors and recipients reported so far, concern entire metabolic pathways related with the production of toxins or the assimilation of nutrients, and the acquisition of detoxification or new metabolic capacities [13], [16]–[18], [60], leading in some cases to a rapid emergence of new pathogenic lineages and to successful host specialization [61]–[63]. The present case fits the previously described scenarios, although it involves a single gene that nevertheless is sufficient to confer fructose-scavenging capabilities to the recipient organism on its own.

Fungi are very often associated with plants, and these are, in turn, the main natural source of fructose, often as a constituent of poly- or oligosaccharides. For example, the metabolism of sucrose (a disaccharide composed of glucose and fructose) plays a very important role in the sugar-partitioning in plant-fungal interactions that occur both in mutualism and pathogenesis [64]. Complex polysaccharides are also a potential source of fructose, and Fsy1 may play a relevant role in the utilization of fructose resulting from degradation of this type of molecule, which will typically yield low concentrations of fructose that can be better assimilated via an uphill transport system. However, both filamentous fungi and yeasts are known to possess other hexose/H^+^ symporters that accept both glucose and fructose (and often other monosacharides) as substrates [64]–[67]. The advantage of having a specific fructose transporter like Fsy1 may lie in the fact that most monosaccharide transporters, including those active at plant/fungal interfaces in mutualistic interactions (mycorrhiza) and pathogenesis, preferentially take up glucose [26], [64]. Consequently, the availability of a specific fructose carrier that is not inhibited by glucose might bring about a considerable increase in efficiency in fructose utilization in environments where both sugars are present. In this respect, it is striking that *O. maius*, the only species among those included in this study that possesses three *FSY1* homologues, is also the only fungus included in the analysis with a primary mycorrhizal lifestyle, suggesting that Fsy1 may be particularly useful in this setting [64]. In addition, given the fact that most Fsy1 homologues described are found in phytopathogenic fungi, it seems plausible that the gene appeared as a specific fructose transporter operating at the plant/fungal interface either in a pathogenic or symbiotic context. This idea should however be revisited once a larger number of genomes in the Ascomycota are available so that the diverse lifestyles are well represented, since there is a bias towards (phyto)pathogenicity in the species sequenced so far.

The highly unusual number of independent HGT involving the *FSY1* gene, the lack of evidence for flanking genes having been co-transferred and the extremely precise “deletions” of the gene in the *A. nidulans clade* and in *E. herbariorum* seem to configure a situation of gains and losses targeting this gene specifically. Another intriguing observation that may have some relation with the above mentioned gene losses in the Eurotiales, is the fact that four species (*A. carbonarius*, *A. aculeatus*, *N. fischeri*, *A. fumigatus*) that retain inter-species synteny upstream of the *FSY1* gene, completely loose synteny immediately downstream of the *FSY1* gene. This suggests that the region in the vicinity of the cognate *FSY1* locus was frequently reused as recombination site and may be particularly prone to genomic instability, at least in this lineage. The reason for this is not obvious, but could lie in the presence of an inconspicuous functional element like an origin of replication, which was previously suggested to favour chromosome fragility [6]. Taken together, these results configure an extraordinarily dynamic evolutionary history centred in a single gene, which is to our knowledge unparalleled in fungi.

Contrarily to the turbulent pattern of gains and losses, there is so far no evidence for marked functional divergence between Fsy1 homologues heterologously expressed in *S. cerevisiae* and spanning large evolutionary distances, both in the Saccharomycotina and the Pezizomycotina [33], [34]. Our results indicate that this also holds for both *A. niger* Fsy1 homologues, so that acquisition of a second gene by HGT does not seem to be related with a function distinct from fructose transport, at least in this case. The *Botryotinia fuckeliana* Fsy1 homologue was shown to mediate fructose uptake in a manner that was not inhibited by glucose, although no kinetic parameters for initial uptake rates are available. On the other hand, expression of the Fsy1 homologue from *Fusarium verticillioides* in *S. cerevisiae* failed to restore fructose transport in a strain devoid of hexose transporters but it is presently unclear whether this could be due to incorrect subcellular localization of the heterologous protein [68]. Finally, it should be noted that all Fsy1 proteins tested so far are also capable of transporting sorbose, albeit to various extents. While this capacity does not seem to be relevant in the context of several Fsy1-harbouring yeast species that do not grow on sorbose, it was probably another important factor influencing Fsy1 evolution in sorbose utilizing fungi, like the yeast *L. starkeyi*.

Taking into account the biochemical properties determined for *Saccharomyces pastorianus* Fsy1, we hypothesize that the signal of the fitness effect imparted by the presence of the gene oscillated in some lineages in the course of evolution, so that *FSY1* gene losses have been fixed at one point in time and later “reversed” by the acquisition of a *FSY1* homologue by HGT. Although this seems the most likely course of events, we cannot exclude that the acquisition of a second phylogenetic distant ortholog by HGT preceded the loss the cognate copy [69]. Intriguingly, in the Pezizomycotina, HGT seems to have been the preferred mechanism to increase the number of *FSY1* genes within a species (like in *A. niger*, *A. kawachii*, *A. brasiliensis*, *A. acidus*, *A. versicolor*, and *O. maius*). On the contrary, in the Saccharomycotina, gene duplications seem to account for all the instances where two genes are found in one species (three *Candida* species and probably in *A. adeninivorans*).

While in prokaryotes events of HGT are very common and the underlying mechanisms are generally well understood, fewer examples of HGT have been reported in fungi and the possible mechanisms involved are largely unknown [20], [58]. In other eukaryotic microbes, HGT has been often associated with endosymbiosis events and with phagocytosis, which are not relevant to explain HGT fungi [5], [20], [58], [70], [71]. Nevertheless, it should be noted that fungi may readily undergo hyphal anastomoses, and that heterokaryon incompatibility usually does not completely prevent cytoplasmic or nuclear exchange [72], [73].

While numerous studies of HGT in fungi were so far based on surveys that detected mainly genes of bacterial origin [10], [74]–[77], this study is centred in a gene encoding a protein with a well-known and unusual phenotype. Most importantly, the *FSY1* gene, being a reasonably recent “invention” in the ascomycetes turned out to be a particularly suitable system to study the dynamics of gene gain and loss while disentangling complicated phenomena like xenolog gene displacement and pseudoparalogy. Orthologous relationships were easy to establish in the Fsy1 cohort and most of the suspected HGT events were confirmed by the expected changes in the chromosomal setting of the gene, in all cases that could be investigated, and were reinforced by topology comparisons.

This study suggests the possibility that the methods used so far to survey large amounts of fungal genomic data to detect HGT may be missing a significant number of intra-kingdom events. Identification and detailed study of such events seems worth pursuing, since they are very likely to provide invaluable new insights in the evolution of eukaryotic genomes.

## Methods

### Preliminary search for putative *FSY1* homologues

To distinguish Fsy1 homologues from other fungal sugar transporters, an initial BLASTP [78] search was performed using the *Saccharomyces pastorianus* Fsy1 protein sequence [GenBank:CAC08232] as query to retrieve putative homologues from GenBank. Sequences with *E*-values lower than 1e-41 were aligned using a fast iterative method in MAFFT v.6.956 and poorly aligned regions were removed using Gblocks v.0.91b [79]. The final alignment consisting of 520 sequences (see Table S3) was used to construct a maximum likelihood (ML) phylogeny in RAxML v7.2.8 [80], using the PROTGAMMAWAG model of amino acid substitution and 100 rapid bootstrap replicates. Sequences with *E*-values lower than 1e-80 formed a well-defined clade representing the entire *FSY1* gene family.

### Database mining for *FSY1* and RNA polymerase homologues

Additional BLASTP and TBLASTN [81] searches were performed to retrieve Fsy1 homologues from the *nr* database in GenBank and from fungal genome databases available as of August 2012. Sequences were retained if their *E*-values were lower than 1e-80 and aligned over the majority of their extension. For *Candida zemplinina* PYCC 3044, about 83 million Illumina paired-end reads were generated with HiSeq 2000 technology and assembled *de novo* using the GS *De Novo* Assembler v.2.6. The final draft assembly consists of 162 scaffolds. We set up a local BLAST database for this genome to search for *FSY1* homologues using the abovementioned criteria. This genome has not been released yet but gene sequences used in this study were deposited in GenBank (Table S1). To construct the species phylogeny (Figure 3), for which whole genome sequence data is available, we used a previously described approach [37]. Briefly, the amino acid sequences of six RNA polymerase subunits (Rpa1, Rpa2, Rpb1, Rpb2, Rpc1, Rpc2) were retrieved from each genome database (including our genome database of *C. zemplinina*) by BLASTP and TBLASTN using *Saccharomyces cerevisiae* RNA polymerase amino acid sequences as query (GenBank:P10964.2, GenBank:P22138.1, GenBank:P04050.2, GenBank:P08518.2, GenBank:P04051.1 and GenBank:P22276.2, respectively). When predicted gene models were unavailable or in cases where proteins were most likely incorrectly predicted, they were re-annotated using AUGUSTUS [82], which relies on a set of training annotation files from several fungal species to offer more precise gene predictions. Fsy1 and RNA polymerase sequences of *Arxula adeninivorans* and *Yarrowia hispaniensis* (*Candida hispaniensis*) were kindly provided by Cécile Neuvéglise (INRA, France). A complete list of fungal taxa, abbreviated species names, genome databases queried in this study and accession numbers of Fsy1 and RNA polymerase proteins is given in Table S1.

### Phylogenetic and comparative topology analyses

Fsy1 and the individual amino acid sequences of six RNA polymerase subunits were aligned using MUSCLE [83] and poorly aligned regions were removed using Gblocks v.0.91b with the following settings: maximum number of contiguous nonconserved positions allowed = 4; minimum length of a block allowed = 10. To construct the species phylogeny (Figure 2), RNA polymerase amino acid sequences were concatenated using Concatenator v.1.1.0 [84] to produce a final dataset of 160 sequences containing 6398 positions. This sequence alignment was subsequently used to construct a maximum likelihood (ML) phylogeny in RAxML v7.2.8 [80], using the PROTGAMMAWAG model. *Rhodotorula graminis*, *Cryptococcus neoformans* and *Ustilago maydis* sequences were used as outgroup. The Fsy1 phylogeny (Figure 1) was also inferred by ML in RAxML using the PROTGAMMAIWAGF model and an alignment of 107 amino acid sequences containing 407 positions. In the absence of a good outgroup the tree was rooted at the midpoint (corresponding to the longest pathway between two operational taxonomical units). Branch supports for both phylogenetic trees were determined using 100 rapid bootstrap replicates. In order to examine the degree of phylogenetic conflict within the RNA polymerase concatenated alignment and in an attempt to corroborate the *FSY1* phylogenetic tree, phylogenetic networks were generated with the same datasets. The alternative splits were found using the NeighborNet method [85], and represented as a phylogenetic network using the Splitstree software v4.12.6 [86], [87]. Comparative topology analyses were performed for each of the putative HGT events that resulted in a phylogeny that conflicts with the species phylogeny. In each case, Fsy1 sequences thought to have been horizontally transferred were constrained to be placed as in the species tree (Figure S4). The Shimodaira-Hasegawa (SH) test [88], as implemented in RAxML, was used to determine whether the ML estimate of each of the constrained topologies differed significantly from the ML estimate of the unconstrained Fsy1 topology (Table S2).

### Evolution of the presence/absence of the *FSY1*

The ancestral state reconstruction of Fsy1 was performed in Mesquite v.2.75 [89]. Discrete characters (presence/absence of *FSY1* gene) were reconstructed using maximum-likelihood [90] with Markov k-state 1 parameter model” (Mk1 est.) [91], which assumes equal probability for changes between states. The species tree inferred from the ML phylogenetic analysis and a character matrix of “absence” or “presence” of Fsy1 in the extant species (scored as binary characters “0” or “1”, respectively), were used as input.

### Comparative syntenic analyses

For each clade, conservation of synteny blocks encompassing *FSY1* was accessed to corroborate orthology and to investigate possible genomic rearrangements. For Saccharomycetes species included in Figure 2, synteny in the vicinity of the *FSY1* gene was inspected using both the Yeast Genome Order Browser (YGOB, http://wolfe.gen.tcd.ie/ygob/) [92] and the Candida Genome Order Browser (CGOB, http://cgob.ucd.ie/) [93], which are online tools for visualizing gene order and context in several yeast genomes available. *FSY1* homologues from *Kluyveromyces lactis* (KLLA0E09021g) and *Candida albicans* (CAWG_01680) were used as query in YGOB and CGOB, respectively. For *Aspergillus* species, ortholog clusters were obtained from the Aspergillus Genome Database (AspGD, http://www.aspergillusgenome.org/) [94] using the two *A. niger FSY1* homologues (An15g01500 and An06g02270) as query. The visualization tool Sybil was used to navigate the ortholog clusters in their genomic context. For *Eurotium herbariorum*, which is not included in the present version of the AspGD, a ∼100 kb annotated region encompassing the *FSY1* homologue (Scaffold 7, 473286–574932) was retrieved from the *E. herbariorium* genome database (http://genome.jgi.doe.gov/Eurhe1/Eurhe1.home.html) and compared using BLAST analyses. For the remaining fungal species, which are not included in these databases, synteny conservation was assessed based on their current annotations as retrieved from their respective genome databases (see Table S1) and confirmed by high scoring BLASTP hits in GenBank.

### Strains used in functional complementation experiments and growth conditions

*S. cerevisiae hxt*-null EBY.VW4000 [50] was used as host for heterologous expression of putative *FSY1* homologues. *Candida albicans* PYCC 3436^T^, *Candida arabinofermentans* PYCC 5603^T^ and *Lipomyces starkeyi* PYCC 4045^T^ were obtained from the Portuguese Yeast Culture Collection (PYCC, Caparica, Portugal) and *Aspergillus niger* ATCC 16404 was obtained from CiiEM, Instituto Superior de Ciências da Saúde Egas, Portugal. Plasmid p415 TEF was obtained from the American Type Culture Collection (ATCC; Manassas, VA, USA). Strains were grown in YPD medium (1% w/v yeast extract, 2% w/v bacto-peptone and 2% w/v glucose) with the exception of *S. cerevisiae* EBY.VW4000 that was grown in YPM medium (1% w/v yeast extract, 2% w/v bacto-peptone and 2% w/v maltose).

### Heterologous expression of *FSY1* homologues in *S. cerevisiae*

Plasmids containing *FSY1* homologues from *S. uvarum* CBS 7001, *C. arabinofermentans* PYCC 5603^T^, *L. starkeyi* PYCC 4045^T^, *C. albicans* PYCC 3436^T^ and both the “yeast-like” and the cognate *FSY1* copies from *A. niger* ATCC 16404 were constructed by homologous recombination in *S. cerevisiae* EBY.VW4000. Intronless *FSY1* homologues were amplified by PCR using genomic DNA isolated as previously described [95]. Two sets of primers and two successive PCR amplifications were performed. In the first PCR, “short” primers matching the 5′ and 3′ ends of *FSY1* coding sequences were used to increase amplicon specificity and yield. The resulting PCR products served as template in a second reaction, which used “long” primers comprising overhangs (38–46 bp) identical to the 3′ end of TEF promoter and the 5′ end of the CYC1 terminator, to allow for homologous recombination into the p415 TEF plasmid. Preparative PCRs were performed in a final volume of 50 µl with the following components: 1X Long PCR buffer with 15 mM MgCl_2_ (Fermentas), 0.20 mM of each of the four deoxynucleoside triphosphates (GE Healthcare), 0.2 µM of each primer, 100–200 ng of genomic DNA, and 2 U Long PCR Enzyme Mix (Fermentas). Thermal cycling consisted of a 3-minute denaturation step at 94°C, followed by an initial round of 10 cycles of denaturation at 94°C for 20 s, annealing for 30 s (variable temperature), extension at 68°C for 2 min, and a second round of 25 cycles increasing the extension time by 1 s in each cycle. A final extension of 10 minutes at 68°C was performed. *FSY1* homologues containing introns (from *C. albicans* and the cognate copy of *A. niger*) were amplified from cDNA. Total RNA was isolated using Trizol (Invitrogen) as previously described [95] from cells grown in Yeast Nitrogen Base (YNB) with 0.5% w/v fructose. First-strand cDNA was synthesized with Super Script III Reverse Transcriptase (Invitrogen) according to the manufacturer's instructions and using the “short” gene-specific reverse primers (see Table S4). The resulting product served as template in a subsequent PCR using the same settings. *S. cerevisiae hxt*-null EBY.VW4000 was transformed [96] simultaneously with p415 TEF linearized with BamHI and HindIII (Roche) and the various gene fragments with the short flanking regions for homologous recombination. Final plasmid constructs, primer sequences and specific annealing temperature are described in Table S4.

### Symport and D-[U-^14^C]fructose uptake assays

Recombinant yeasts harboring *FSY1* homologues were grown in liquid YNB (without aminoacids) medium with 1% w/v fructose containing uracil, tryptophan and histidine. Cells were grown to mid exponential phase (OD_640_ between 0.8 and 1.2), harvested by centrifugation, washed twice with sterile cold water and resuspended to a final concentration of 20 to 22 mg dry weight/ml. The presence of symport activity was assessed by computer recording the alkalization of an aqueous yeast cell suspension elicited by the addition of fructose or sorbose, using a standard pH meter [65] and a home designed software. The absence of glucose transport was confirmed by the absence of acidification after sugar addition to the cell suspension. Transport of D-[U-^14^C]fructose was measured according to the procedures described by Spencer-Martins and Van Uden [97]. Kinetic parameters were determined by non-linear regression (Michaelis-Menten Equation) using GraphPad Prism (v5.00 for Windows, GraphPad Software, San Diego California USA).

## Supporting Information

Figure S1 Phylogenetic tree depicting the relationship between Fsy1 homologues and other fungal transporter proteins. The ML phylogeny separates the Fsy1 homologues (highlighted in orange) from the other putative sugar transporters, most of which are still uncharacterized. The ‘gi’ (GenInfo Identifier) number was assigned for proteins other than Fsy1 homologues and their complete names/GenBank accession numbers are given in Table S3. Itr1 and Itr2 are two myo-inositol transporters previously characterized in *Saccharomyces cerevisiae*. *E*-values resulting from BLASTP analysis (see methods) are shown for sequences indicated by a star. The highest *E*-value found for a Fsy1 homologue was 1e-81. Bootstrap support values are depicted in tree branches (>50%) as given in the key. Sequences are colored according to their phylogenic lineage (red, Saccharomycotina; blue, Pezizomycotina; Purple, Taphrinomycotina).
(TIF)

Figure S2 Gene content and organization in *FSY1* loci in Pezizomycotina. Chromosomal regions (chr) or scaffolds (sc) encompassing *FSY1* gene are depicted by grey bars for most of the species represented in the species tree (of which a subsection is shown on the left). Green bars represent regions where *FSY1* gene is located and likely acquired by HGT. The *FSY1* gene is shown as a red arrow, denoting transcriptional orientation. Within each clade, highlighted by a different background color, orthologous genes exhibit the same color. Non-syntenic genes, Ty elements and uncertainly annotated genes are colored in grey, black and white, respectively. The end of a chromosome/scaffold is indicated by a bracket next to a gene. Genomic locus or accession numbers are shown for each gene as they appear in their respective genome databases. Species names are abbreviated as given in Table S1.
(TIF)

Figure S3 Species phylogenetic network. The network was constructed using the same concatenated alignment of six RNA polymerase amino acid sequences as used for constructing the species tree (see methods). The neighbor-net method was used to infer splits. Saccharomycotina and Pezizomycotina clades are colored as in Figures 1 and 3. The topology of the species tree (Figure 2) and phylogenetic network are mostly congruent, except for the position of *Ascoidea rubescens*. Species names are abbreviated as in Table S1.
(TIF)

Figure S4 Topology tests for each of the presumed HGT events involving Fsy1. (A) Unconstrained Fsy1 ML tree topology emphasizing each of the presumed HGT events (numbered) as discussed in the text. Background colors in the species abbreviated names refer to taxa to which each species belongs (see Figures 1 and 3 for comparison). (B) Constrained topologies for each of the putative HGT events where Fsy1 sequences (highlighted in branches) were forced to conform to the species tree. The tree topology 11a differs from the 11b since in the former the protein sequences Milfar1 and Milfar2, which are associated with Aspergilli in the Fsy1 ML topology, were also included in the constraint compatible with monophyly of CUG clade species.
(TIF)

Figure S5 Fsy1 phylogenetic network. The network was constructed using the Fsy1 tree dataset (Figure 1) and inferred using the neighbor-net method. Clades are colored as in Figures 1 and 3. The Fsy1 network and Fsy1 tree have comparable topologies, which reinforce the putative HGT events detected. Species names are abbreviated as in Table S1.
(TIF)

Figure S6 Comparative analysis depicting the annotation of syntenic and non-syntenic genes in the *FSY1* homolog genomic region in Eurotiales. Syntenic region centered on the gene located immediately upstream (panel A) or downstream (panel B) of the *FSY1* gene in *Aspergillus niger* (“hyp-MFS” and “*NEO1*”, respectively). In each panel, the gene name enclosed in a box was used as query in the AspGD. Gross structural rearrangements such as (i) an insertion in *Aspergillus aculeatus* and (ii) an inversion in *Aspergillus fumigatus* and *Aspergillus carbonarius* are highlighted in yellow and orange background colors, respectively. All the remaining features are as in Figure 4.
(TIF)

Figure S7 Genomic location of *FSY1* gene in *Aspergillus versicolor* and comparison with the homologous region in *A. nidulans* and *A. sydowii*. Orthologs are connected by grey or pink bars depending on their relative orientation (pink bars depict inversions). Synteny is highly conserved between *A. versicolor* and *A. sydowii*, except for the presence of *FSY1* gene. Between *A. versicolor* and *A. nidulans* several gene rearrangements can be observed.
(TIF)

Table S1 Complete list of fungal taxa, abbreviated species names, genome databases used for BLAST searches and accession numbers of Fsy1 and RNA polymerase proteins. Different groups of fungi are shown in different colors (Green: “early diverging fungal lineages”; pink: Basidiomycota; blue: Ascomycota). Abbreviated species names (Abb.) are given for each species. RNA polymerase subunit amino acid sequence accession numbers are shown only for species used to construct the species tree. For sequences retrieved from JGI databases, the Protein ID number is given. ^a^original protein prediction was modified to correct error. ^b^sequence spanned multiple contigs. ^c^partial sequence not used. ^d^partial sequence. ‘n.d.’ indicates *FSY1* homologues ‘not detected’ by BLASTP or TBLASTN. ‘SSS website’ Genome database stands for “Saccharomyces sensu stricto (SSS) Website” (http://www.saccharomycessensustricto.org/cgi-bin/s3).
(PDF)

Table S2 Constraint analysis for each of the presumed HGT events involving Fsy1. The Shimodaira-Hasegawa (SH) test as implemented in RAxML was used to compare Fsy1 constrained topologies with the Fsy1 ML topology. *P* values were considered significant if less than 0.05.
(PDF)

Table S3 List of protein sequences used to construct the phylogenetic tree in Figure S1. Protein sequences were retrieved from GenBank using BLASTP searches (see methods). Accession numbers of Fsy1 homologues are given in Table S1.
(XLS)

Table S4 List of primers, specific PCR conditions, plasmid constructs and strains used for heterologous expression of *FSY1* homologues in *S. cerevisiae hxt*-null. Primer sequences and annealing temperatures used to amplify each *FSY1* homologue are indicated. Plasmids containing *FSY1* homologues derived from the indicated fungal species were constructed by homologous recombination in *S. cerevisiae* EBY.VW4000. ^a^primer used for first-strand cDNA synthesis. ^b^primers with overhangs identical to the 3′ end of TEF promoter (underlined) and the 5′ end of the CYC1 terminator (bold) used for homologous recombination into the p415 TEF plasmid. ^T^ Type strain.
(PDF)
