# Supplementary figures and images for: Extensive Intra-Kingdom Horizontal Gene Transfer Converging on a Fungal Fructose Transporter Gene
Source: PLoS Genet. 2013 Jun 20;9(6):e1003587. doi: 10.1371/journal.pgen.1003587 (PMC3688497; doi:10.1371/journal.pgen.1003587)

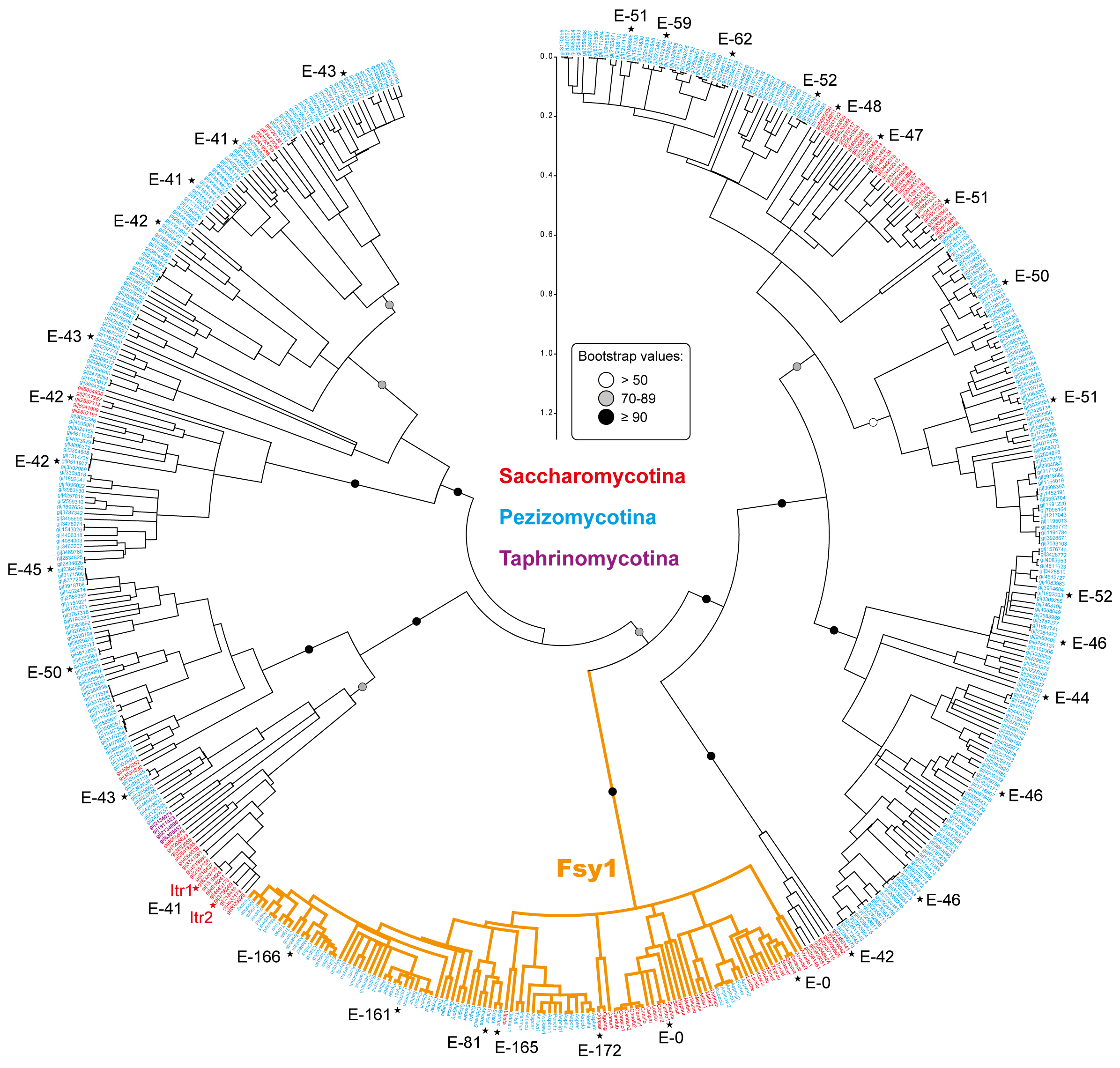

Supplement: Figure S1 — Phylogenetic tree depicting the relationship between Fsy1 homologues and other fungal transporter proteins. The ML phylogeny separates the Fsy1 homologues (highlighted in orange) from the other putative sugar transporters, most of which are still uncharacterized. The ‘gi’ (GenInfo Identifier) number was assigned for proteins other than Fsy1 homologues and their complete names/GenBank accession numbers are given in Table S3. Itr1 and Itr2 are two myo-inositol transporters previously characterized in Saccharomyces cerevisiae. E-values resulting from BLASTP analysis (see methods) are shown for sequences indicated by a star. The highest E-value found for a Fsy1 homologue was 1e-81. Bootstrap support values are depicted in tree branches (>50%) as given in the key. Sequences are colored according to their phylogenic lineage (red, Saccharomycotina; blue, Pezizomycotina; Purple, Taphrinomycotina). (TIF) [file pgen.1003587.s001.tif]

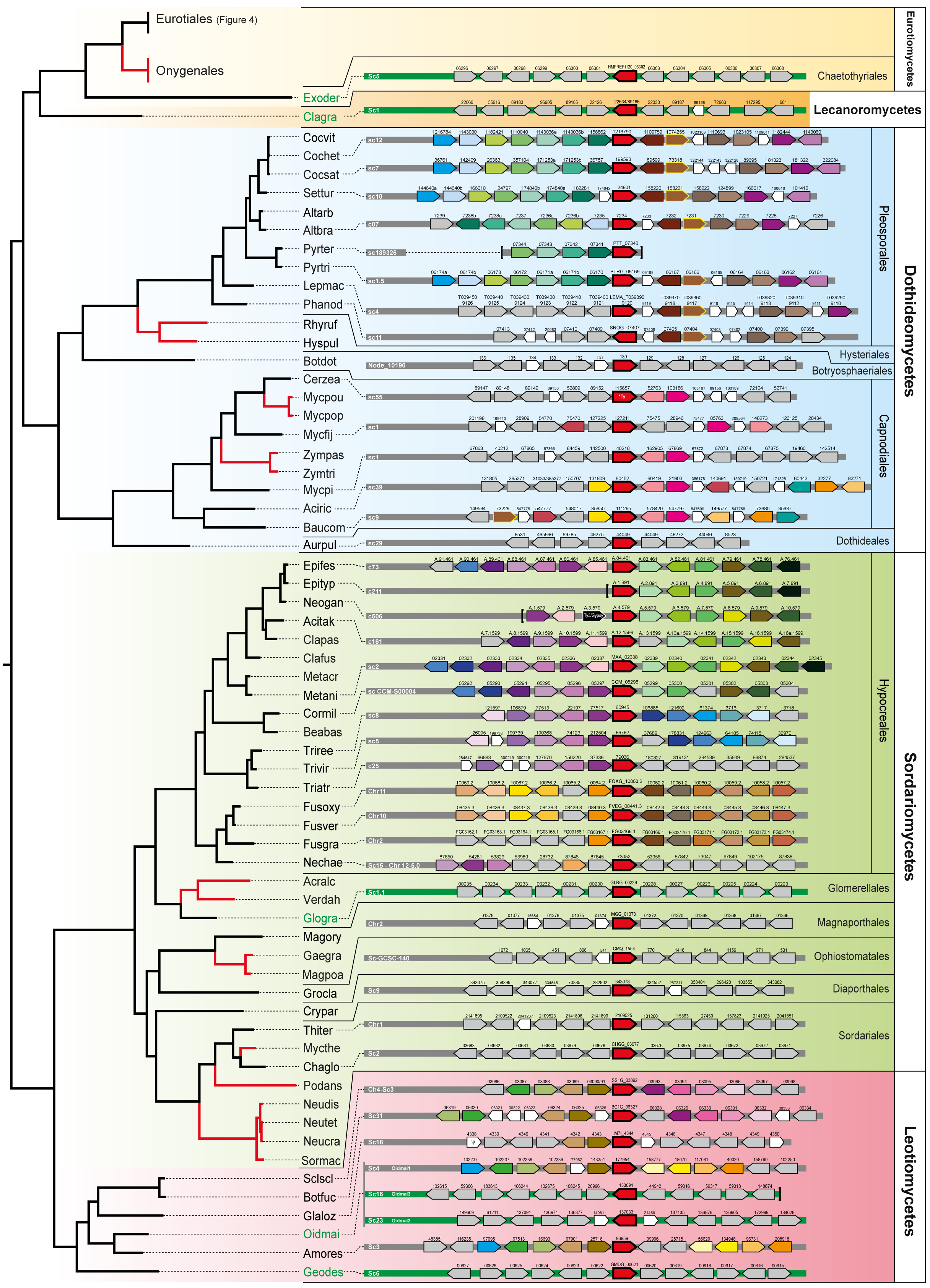

Supplement: Figure S2 — Gene content and organization in FSY1 loci in Pezizomycotina. Chromosomal regions (chr) or scaffolds (sc) encompassing FSY1 gene are depicted by grey bars for most of the species represented in the species tree (of which a subsection is shown on the left). Green bars represent regions where FSY1 gene is located and likely acquired by HGT. The FSY1 gene is shown as a red arrow, denoting transcriptional orientation. Within each clade, highlighted by a different background color, orthologous genes exhibit the same color. Non-syntenic genes, Ty elements and uncertainly annotated genes are colored in grey, black and white, respectively. The end of a chromosome/scaffold is indicated by a bracket next to a gene. Genomic locus or accession numbers are shown for each gene as they appear in their respective genome databases. Species names are abbreviated as given in Table S1. (TIF) [file pgen.1003587.s002.tif]

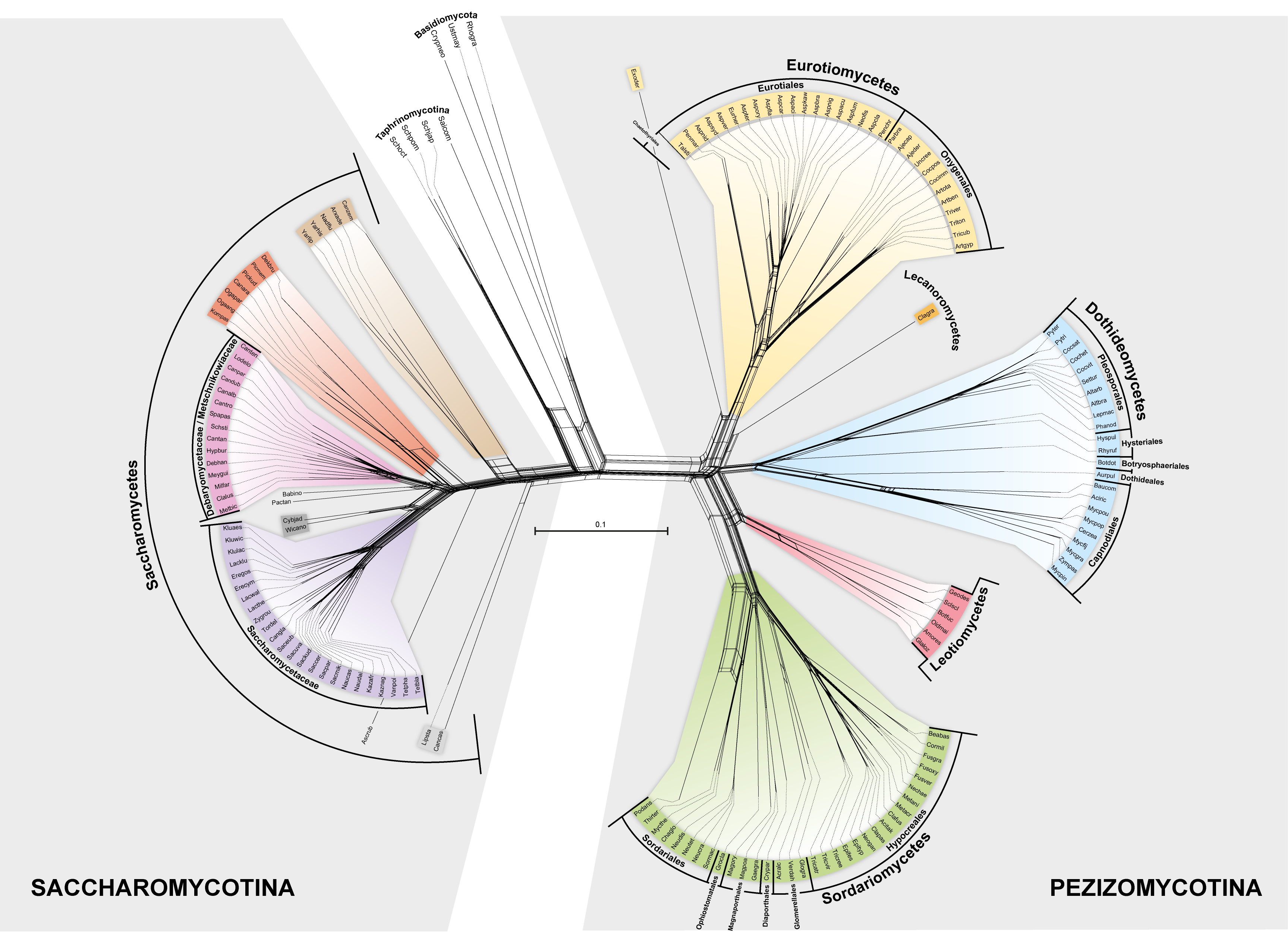

Supplement: Figure S3 — Species phylogenetic network. The network was constructed using the same concatenated alignment of six RNA polymerase amino acid sequences as used for constructing the species tree (see methods). The neighbor-net method was used to infer splits. Saccharomycotina and Pezizomycotina clades are colored as in Figures 1 and 3. The topology of the species tree (Figure 2) and phylogenetic network are mostly congruent, except for the position of Ascoidea rubescens. Species names are abbreviated as in Table S1. (TIF) [file pgen.1003587.s003.tif]

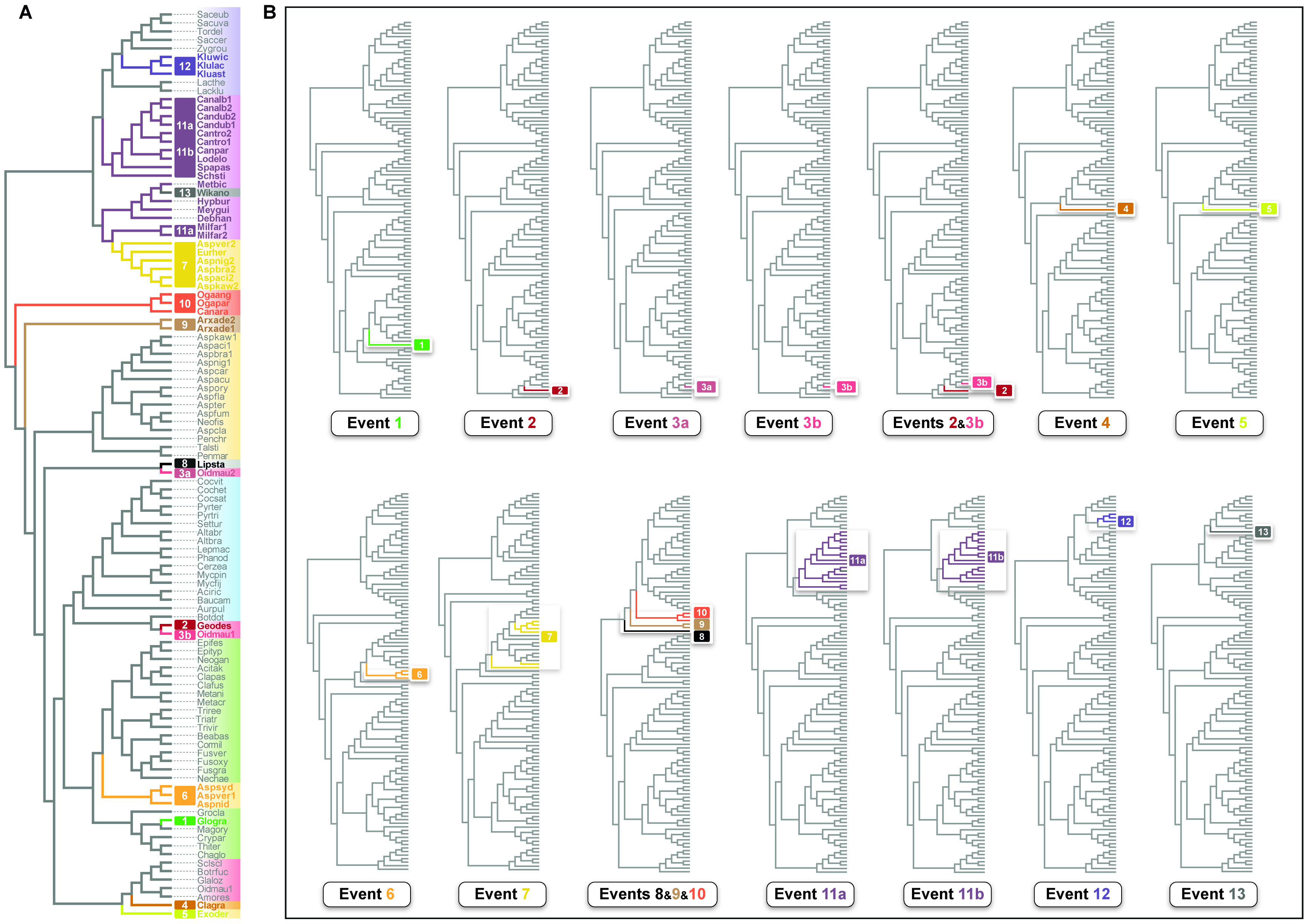

Supplement: Figure S4 — Topology tests for each of the presumed HGT events involving Fsy1. (A) Unconstrained Fsy1 ML tree topology emphasizing each of the presumed HGT events (numbered) as discussed in the text. Background colors in the species abbreviated names refer to taxa to which each species belongs (see Figures 1 and 3 for comparison). (B) Constrained topologies for each of the putative HGT events where Fsy1 sequences (highlighted in branches) were forced to conform to the species tree. The tree topology 11a differs from the 11b since in the former the protein sequences Milfar1 and Milfar2, which are associated with Aspergilli in the Fsy1 ML topology, were also included in the constraint compatible with monophyly of CUG clade species. (TIF) [file pgen.1003587.s004.tif]

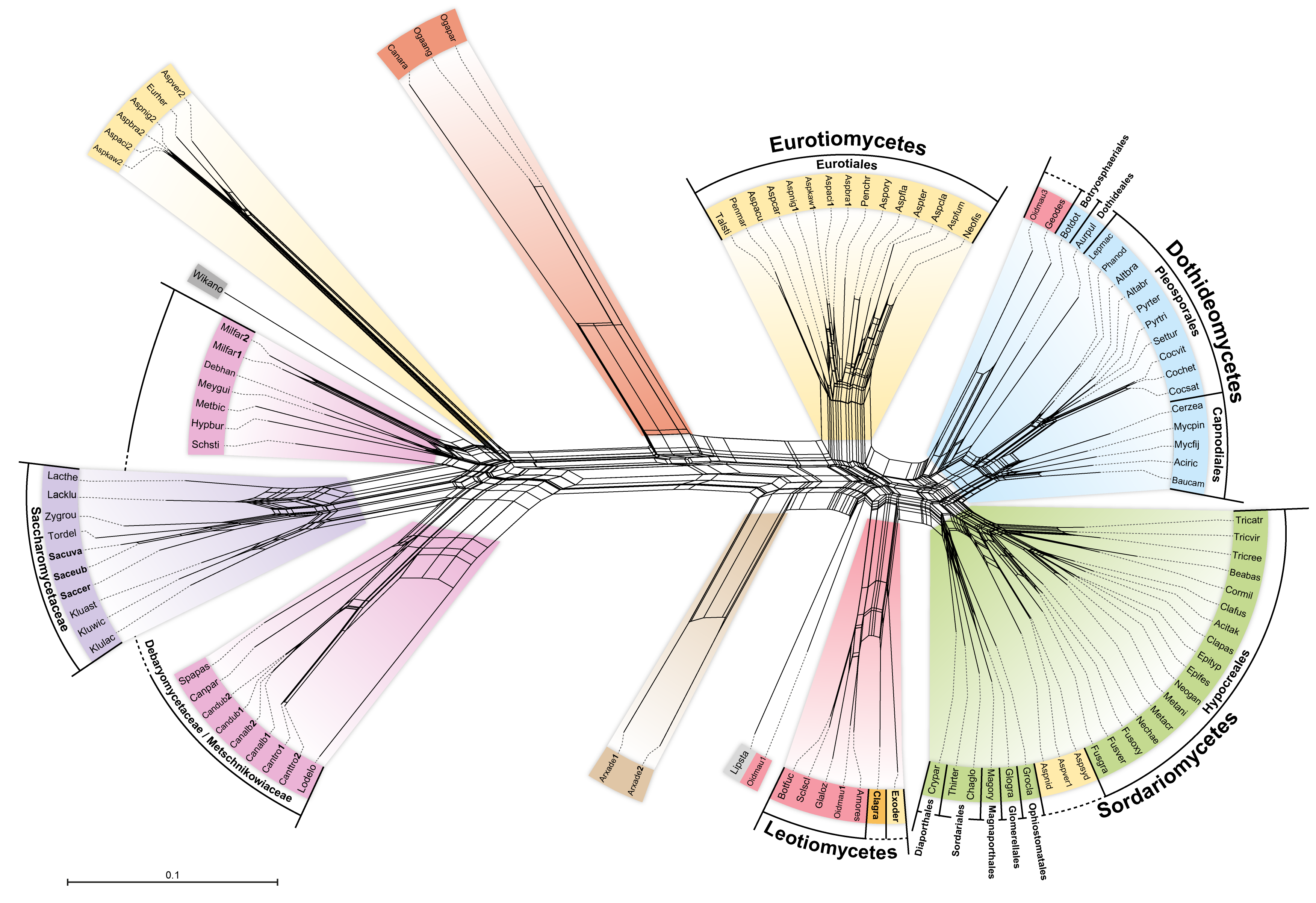

Supplement: Figure S5 — Fsy1 phylogenetic network. The network was constructed using the Fsy1 tree dataset (Figure 1) and inferred using the neighbor-net method. Clades are colored as in Figures 1 and 3. The Fsy1 network and Fsy1 tree have comparable topologies, which reinforce the putative HGT events detected. Species names are abbreviated as in Table S1. (TIF) [file pgen.1003587.s005.tif]

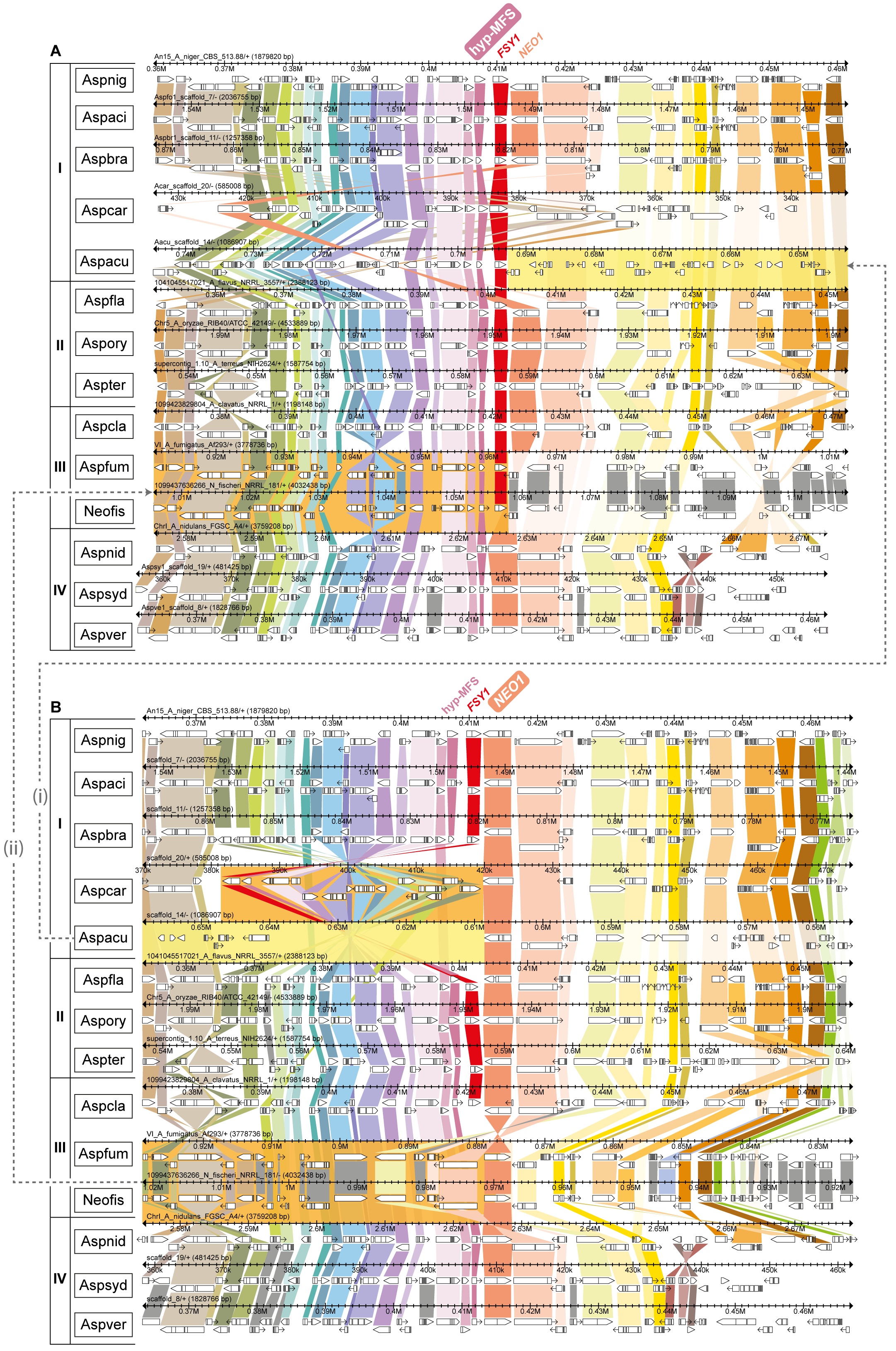

Supplement: Figure S6 — Comparative analysis depicting the annotation of syntenic and non-syntenic genes in the FSY1 homolog genomic region in Eurotiales. Syntenic region centered on the gene located immediately upstream (panel A) or downstream (panel B) of the FSY1 gene in Aspergillus niger (“hyp-MFS” and “NEO1”, respectively). In each panel, the gene name enclosed in a box was used as query in the AspGD. Gross structural rearrangements such as (i) an insertion in Aspergillus aculeatus and (ii) an inversion in Aspergillus fumigatus and Aspergillus carbonarius are highlighted in yellow and orange background colors, respectively. All the remaining features are as in Figure 4. (TIF) [file pgen.1003587.s006.tif]

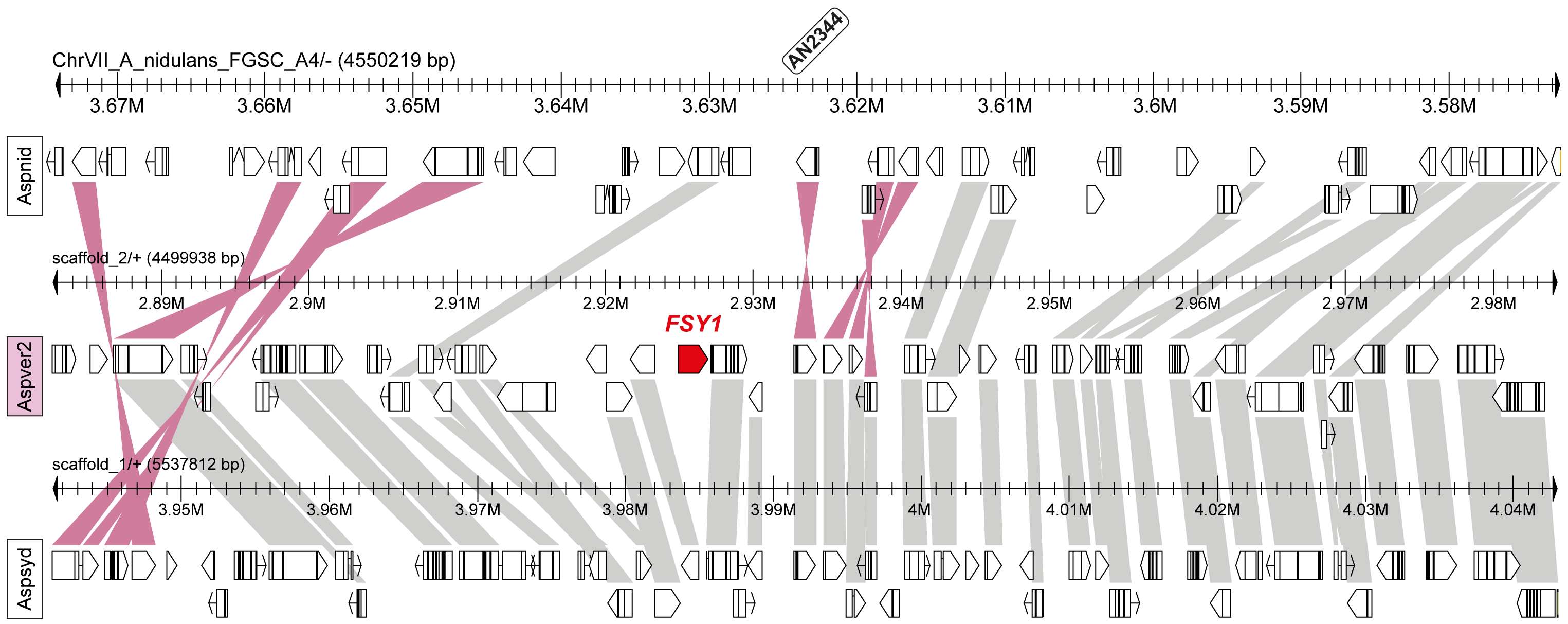

Supplement: Figure S7 — Genomic location of FSY1 gene in Aspergillus versicolor and comparison with the homologous region in A. nidulans and A. sydowii. Orthologs are connected by grey or pink bars depending on their relative orientation (pink bars depict inversions). Synteny is highly conserved between A. versicolor and A. sydowii, except for the presence of FSY1 gene. Between A. versicolor and A. nidulans several gene rearrangements can be observed. (TIF) [file pgen.1003587.s007.tif]
