# Supplementary material for: Extensive Intra-Kingdom Horizontal Gene Transfer Converging on a Fungal Fructose Transporter Gene
Source: PLoS Genet. 2013 Jun 20;9(6):e1003587. doi: 10.1371/journal.pgen.1003587 (PMC3688497; doi:10.1371/journal.pgen.1003587)
Supplement: Table S1 — Complete list of fungal taxa, abbreviated species names, genome databases used for BLAST searches and accession numbers of Fsy1 and RNA polymerase proteins. Different groups of fungi are shown in different colors (Green: “early diverging fungal lineages”; pink: Basidiomycota; blue: Ascomycota). Abbreviated species names (Abb.) are given for each species. RNA polymerase subunit amino acid sequence accession numbers are shown only for species used to construct the species tree. For sequences retrieved from JGI databases, the Protein ID number is given. aoriginal protein prediction was modified to correct error. bsequence spanned multiple contigs. cpartial sequence not used. dpartial sequence. ‘n.d.’ indicates FSY1 homologues ‘not detected’ by BLASTP or TBLASTN. ‘SSS website’ Genome database stands for “Saccharomyces sensu stricto (SSS) Website” (http://www.saccharomycessensustricto.org/cgi-bin/s3). (PDF) [file pgen.1003587.s008.pdf]

Table S1

Phylum/ sub-phylum	Species	Abb.	Strain(s)	Genome database	Fsy1 (E < 1e-80)	Rpa1	Rpa2	Rpb1	Rpb2	Rpc1	Rpc2
Blastocladiomycota	*Allomyces macrogynus*		ATCC 38327	BROAD	n.d.						
Blastocladiomycota	*Catenaria anguillulae*		PL171 (v1.0)	JGI	n.d.						
Chytridiomycota	*Batrachochytrium dendrobatidi*		JAM81 (v1.0)<br>JEL423	JGI<br>BROAD	n.d.<br>n.d.						
Chytridiomycota	*Gonapodya prolifera*		JEL478 (v1.0)	JGI	n.d.						
Chytridiomycota	*Homoloaphlyctis polyrhiza*		JEL 142	NCBI	n.d.						
Chytridiomycota	*Spizellomyces punctatus*		DAOM BR117	BROAD	n.d.						
Entomophthoromycotina	*Conidiobolus coronatus*		NRRL 28638	JGI	n.d.						
Glomeromycota	*Glomus intraradices* (EST data)			INRA/JGI	n.d.						
Kickxellomycotina	*Coemansia reversa*		NRRL 1564 (v.10)	JGI	n.d.						
Microsporidia	*Edhazardia aedis*		USNM 41457	BROAD/NCBI	n.d.						
Microsporidia	*Encephalitozoon cuniculi*		GB-M1<br>EC1	Genoscope/NCBI<br>BROAD/NCBI	n.d.						
Microsporidia	*Encephalitozoon intestinalis*		ATCC 50506	NCBI	n.d.						
Microsporidia	*Enterocytozoon bieneusi*		H348	NCBI	n.d.						
Microsporidia	*Hamiltosporidium tvaerminnensis*		OER-3-3	NCBI	n.d.						
Microsporidia	*Nematocida parisii*		ERTm1<br>ERTm3	BROAD/NCBI<br>BROAD/NCBI	n.d.<br>n.d.						
Microsporidia	*Nosema ceranae*		BRL01	NCBI	n.d.						
Microsporidia	*Vavraia culicis 'floridensis'*			BROAD/NCBI	n.d.						
Microsporidia	*Vittaforma corneae*		ATCC 50505	BROAD/NCBI	n.d.						
Mortierellomycotina	*Mortierella verticillata*		NRRL 6337	BROAD	n.d.						
Mucoromycotina	*Mucor circinelloides*		CBS277.49 (v2.0)	JGI	n.d.						
Mucoromycotina	*Phycomyces blakesleeanus*		NRRL1555 (v2.0)	JGI	n.d.						
Mucoromycotina	*Rhizopus oryzae*		RA 99-880	BROAD	n.d.						
Neocallimastigomycota	*Piromyces sp.*		E2 (v1.0)	JGI	n.d.						
Agaricomycotina	*Agaricus bisporus var. bisporus*		H97 (v2.0)	JGI	n.d.						
Agaricomycotina	*Agaricus bisporus var. burnettii*		JB137-S8	JGI	n.d.						
Agaricomycotina	*Auricularia delicata*		TFB-10046 (v1.0)	JGI	n.d.						
Agaricomycotina	*Bjerkandera adusta*		SB-22 (v1.0)	JGI	n.d.						
Agaricomycotina	*Botryobasidium botryosum*		FD-172 (v1.0)	JGI	n.d.						

Phylum/ sub-phylum	Species	Abb.	Strain(s)	Genome database	Fsy1 (*E* < 1e-80)	Rpa1	Rpa2	Rpb1	Rpb2	Rpc1	Rpc2
Agaricomycotina	*Ceriporiopsis subvermispora*		B	JGI	n.d.						
Agaricomycotina	*Coniophora puteana*		RWD-64-598 (v1.0)	JGI	n.d.						
Agaricomycotina	*Coprinopsis cinerea*		okayama7#130	BROAD	n.d.						
Agaricomycotina	*Cryptococcus gatti*		WM276	NCBI	n.d.						
Agaricomycotina	*Cryptococcus neoformans* var. *grubii*		H99	BROAD	n.d.						
Agaricomycotina	*Cryptococcus neoformans var. neoformans*	*Cryneo*	JEC21<br>B-3501A	NCBI	n.d.	XP_570516	XP_571380	XP_570943	XP_570204	XP_571468	XP_572718
Agaricomycotina	*Dacryopinax* sp.		DJM-731 (v1.0)	JGI	n.d.						
Agaricomycotina	*Dichomitus squalens*		LYAD-421 (v1.0)	JGI	n.d.						
Agaricomycotina	*Fibroporia radiculosa*		TFFH 294	NCBI	n.d.						
Agaricomycotina	*Fomitiporia mediterranea*		MF3/22 (v1.0)	JGI	n.d.						
Agaricomycotina	*Fomitopsis pinicola*		FP-58527 (v1.0)	JGI	n.d.						
Agaricomycotina	*Ganoderma lucidum*		G.260125-1	NCBI	n.d.						
Agaricomycotina	*Ganoderma* sp.		10597 SS1 (v1.0)	JGI	n.d.						
Agaricomycotina	*Gloeophyllum trabeum*		ATCC 11539 (v1.0)	JGI	n.d.						
Agaricomycotina	*Gymnopus luxurians*		FD-317 M1 (v1.0)	JGI	n.d.						
Agaricomycotina	*Hebeloma cylindrosporum*		h7 (v1.0)	JGI	n.d.						
Agaricomycotina	*Heterobasidion irregulare*		TC32-1 (v2.0)	JGI	n.d.						
Agaricomycotina	*Hydnomerulius pinastri*		MD-312 (v2.0)	JGI	n.d.						
Agaricomycotina	*Hypholoma sublateritium*		FD-334 SS-4 (v1.0)	JGI	n.d.						
Agaricomycotina	*Jaapia argillacea*		MUCL 33604 (v1.0)	JGI	n.d.						
Agaricomycotina	*Laccaria bicolor*		S238N-H82 (v2.0)	JGI	n.d.						
Agaricomycotina	*Moniliophthora perniciosa*		FA553	NCBI	n.d.						
Agaricomycotina	*Paxillus involutus*		ATCC200175 (v1.0)	JGI	n.d.						
Agaricomycotina	*Phanerochaete chrysosporium*		RP-78 (v2.0)	JGI	n.d.						
Agaricomycotina	*Phlebia brevispora*		HHB-7030 (v1.0)	JGI	n.d.						
Agaricomycotina	*Phlebiopsis gigantea*		11061_1 (v1.0)	JGI	n.d.						
Agaricomycotina	*Piloderma croceum*		F 1598 (v1.0)	JGI	n.d.						
Agaricomycotina	*Piriformospora indica*		DSM 11827	NCBI	n.d.						
Agaricomycotina	*Pisolithus microcarpus*		441 (v1.0)	JGI	n.d.						
Agaricomycotina	*Pisolithus tinctorius*		Marx 270 (v1.0)	JGI	n.d.						
Agaricomycotina	*Pleurotus ostreatus*		PC15 (v2.0)<br>PC9 (v1.0)	JGI	n.d.						

**Phylum/<br>sub-phylum**	**Species**	**Abb.**	**Strain(s)**	**Genome database**	**Fsy1**<br>(E < 1e-80)	**Rpa1**	**Rpa2**	**Rpb1**	**Rpb2**	**Rpc1**	**Rpc2**
Agaricomycotina	*Plicaturopsis crispa*		(v1.0)	JGI	n.d.						
Agaricomycotina	*Postia placenta*		Mad-698-R (v1.0)	JGI	n.d.						
Agaricomycotina	*Punctularia strigosozonata*		HHB-11173 SS5	JGI	n.d.						
Agaricomycotina	*Schizophyllum commune*		H4-8 (v2.0)	JGI	n.d.						
Agaricomycotina	*Serpula lacrymans var. lacrymans*		S7.3 (v2.0)	JGI	n.d.						
Agaricomycotina	*Sphaerobolus stellatus*		SS14 (v1.0)	JGI	n.d.						
Agaricomycotina	*Stereum hirsutum*		FP-9166 (v1.0)	JGI	n.d.						
Agaricomycotina	*Trametes versicolor*		FP-101664 (v1.0)	JGI	n.d.						
Agaricomycotina	*Tremella mesenterica*		DSM 1558 (v1.0)	JGI/NCBI	n.d.						
Agaricomycotina	*Wallemia sebi*		CBS 633.66								
Agaricomycotina	*Wolfiporia cocos*		MD-104 SS10 (v1.0)	JGI	n.d.						
Pucciniomycotina	*Melampsora laricis-populina*		98AG31 (v1.0)	JGI	n.d.						
Pucciniomycotina	*Microbotryum violaceum*		p1A1 Lamole	BROAD	n.d.						
Pucciniomycotina	*Mixia osmundae*		IAM 14324 (v1.0)		n.d.						
Pucciniomycotina	*Puccinia graminis* f. sp. *tritici*		CRL 75-36-700-3	BROAD	n.d.						
Pucciniomycotina	*Puccinia triticina*		1-1 BBBD Race 1	BROAD	n.d.						
Pucciniomycotina	*Rhodosporidium toruloides*		MTCC 457	NCBI	n.d.						
Pucciniomycotina	*Rhodotorula glutinis*		ATCC 204091	NCBI	n.d.						
Pucciniomycotina	*Rhodotorula graminis*	*Rhogra*	WP1 (v1.1)	JGI	n.d.	25654	32812	54819	56094	33526	39933
Pucciniomycotina	*Sporobolomyces* sp.		IAM13481 (v1.0)	JGI	n.d.						
Ustilaginomycotina	*Malassezia globosa*		CBS 7966	NCBI/ P&G	n.d.						
Ustilaginomycotina	*Malassezia restricta*		CBS 7877	NCBI/ P&G	n.d.						
Ustilaginomycotina	*Ustilago hordei*		Uh4875-4	BROAD/NCBI	n.d.						
Ustilaginomycotina	*Ustilago maydis*	*Ustmay*	521<br>FB1	BROAD/NCBI<br>JGI	n.d.	EAK83798	EAK81692	EAK84769	2446[a]	EAK87041	EAK83484
Pezizomycotina	*Aciculosporium take*	*Acitak*	MAFF-241224	NCBI	Contig00161	AFQZ01001422.1	AFQZ01000020.1	AFQZ01000109.1	AFQZ01000184.1	AFQZ01000682.1	AFQZ01000110.1
Pezizomycotina	*Acidomyces richmondensis*	*Aciric*	BFW	JGI	60452	356353	70916	4084	149678	540	60517
Pezizomycotina	*Acremonium alcalophilum*	*Acralc*	JCM 7366	JGI	n.d.	1050875	1049371	1093047[a]	1062944	1075933	1079193
Pezizomycotina	*Ajellomyces capsulatus (Histoplasma capsulatum)*	*Ajecap*	NAm1	BROAD/NCBI	n.d.	XP_001539109	XP_001538304	XP_001542193	XP_001540601	XP_001536231	EEH02917.1
Pezizomycotina	*Ajellomyces dermatitidis*	*Ajeder*	SLH14081	BROAD/NCBI	n.d.	EGE80034.1	XP_002626186.1	XP_002621402.1	XP_002624828.1	EEQ91497.1	XP_002623660.1
Pezizomycotina	*Alternaria arborescens*	*Altarb*	EGS 39-128	NCBI	NODE_148	AIIC01000010.1	AIIC01000068.1	AIIC01000024.1	AIIC01000055.1	AIIC01000003.1	AIIC01000009.1
Pezizomycotina	*Alternaria brassicicola*	*Altbra*	ATCC 96836	JGI/NCBI	7234	8388 (d)	9486	3233[a,d]	3555	6953	6038 (a)

Phylum/ sub-phylum	Species	Abb.	Strain(s)	Genome database	Fsy1 (E < 1e-80)	Rpa1	Rpa2	Rpb1	Rpb2	Rpc1	Rpc2
Pezizomycotina	*Amorphotheca resinae*	*Amores*	ATCC 22711	JGI	96895	98634	141813	110089	100240	130891	69614
Pezizomycotina	*Arthroderma benhamiae*	*Artben*	CBS 112371	BROAD/NCBI	n.d.	ARB_00090[a]	ARB_07934	ARB_00027[a]	ARB_06116[a]	ARB_01618	ARB_07068
Pezizomycotina	*Arthroderma gypseum*	*Artgyp*	CBS 118893	BROAD/NCBI	n.d.	XP_003177534.1	XP_003169207.1	XP_003177452.1	XP_003170144.1	XP_003177700.1	XP_003173614.1
Pezizomycotina	*Arthroderma otae* *(Microsporum canis)*	*Artota*	CBS 113480	BROAD/NCBI	n.d.	XP_002850253.1	XP_002842776.1	XP_002850330.1	XP_002849456.1	XP_002851111.1	XP_002844863.1
Pezizomycotina	*Aspergillus acidus (foetidus)*	*Aspaci*	CBS 106.47	JGI	61430 211516	31515	173651	204767	37284[a]	143391[a]	39857
Pezizomycotina	*Aspergillus aculeatus*	*Aspacu*	ATCC 16872	JGI	80714	Scaffold1	Scaffold3	Scaffold1	Scaffold17	Scaffold20	Scaffold6
Pezizomycotina	*Aspergillus brasiliensis*	*Aspbra*	CBS 101740	JGI	132411 201768	505552	241815	39443	35001[a]	209077	466230
Pezizomycotina	*Aspergillus carbonarius*	*Aspcar*	ITEM 5010	JGI	9976	Scaffold1	204799	507279	211598	208763[a]	207237
Pezizomycotina	*Aspergillus clavatus*	*Aspcla*	NRRL 1	NCBI	XP_001270153	XP_001268868.1	XP_001267972.1	XP_001268791.1	XP_001272355.1	XP_001271388.1	XP_001269884.1
Pezizomycotina	*Aspergillus flavus*	*Aspfla*	NRRL 3357	NCBI	XP_002378351	XP_001727495.1	XP_001819329.1	XP_002374837.1	XP_002380667.1	XP_002383676.1	XP_002373016.1
Pezizomycotina	*Aspergillus fumigatus*	*Aspfum*	Af293	NCBI	XP_001481451	XP_752760.1	XP_750719.1	XP_752837.1	XP_746740.1	XP_754189.1	XP_749996.1
Pezizomycotina	*Aspergillus kawachii*	*Aspkaw*	IFO 4308	NCBI	GAA90630 GAA91467	GAA87457.1	GAA84045.1	GAA86711.1	GAA91151.1	GAA88313.1	GAA83509.1
Pezizomycotina	*Aspergillus nidulans*	*Aspnid*	FGSC A4	BROAD/NCBI	XP_660398	ANID_12215.1[d]	XP_661537.1	XP_658413.1	CBF82508.1	CBF87003.1	XP_657925.1
Pezizomycotina	*Aspergillus niger*	*Aspnig*	CBS 513.88	BROAD/JGI	XP_001396690 XP_001391049	EHA18239.1	CAK45911.1	EHA27022.1	XP_001395161.2	XP_001393726.1	XP_001388998.2
Pezizomycotina	*Aspergillus oryzae*	*Aspory*	RIB40	NCBI	XP_001823028	XP_001727495.1	XP_001819329.1	XP_002374837.1	XP_002380667.1	XP_001824913.1	XP_001817863.2
Pezizomycotina	*Aspergillus sydowii*	*Aspsyd*		JGI	43335	86485	150494	139296	50074	56036[a]	85930[a]
Pezizomycotina	*Aspergillus terreus*	*Aspter*	NIH 2624	BROAD/NCBI	XP_001209739	XP_001210679.1	XP_001215005.1	XP_001210766.1	XP_001209185.1	XP_001212669.1	XP_001214023.1
Pezizomycotina	*Aspergillus versicolor*	*Aspver*	CBS 583.65	JGI	40258 38376	23862	665103	78733	47803	51108[a]	48573[a]
Pezizomycotina	*Aureobasidium pullulans var. subglaciale*	*Aurpul*	EXF-2481	JGI	44049	61973	104710	109539	6397	7571	400562
Pezizomycotina	*Baudoinia compniacensis*	*Baucom*	UAMH 10762	JGI	111295	34468	34698	85149	103650	78328	156724
Pezizomycotina	*Beauveria bassiana*	*Beabas*	ARSEF 2860	NCBI	BBA_S00015_3	ADAH01000331.1	ADAH01000478.1	ADAH01000013.1	ADAH01001056.1	ADAH01000063.1	ADAH01000357.1
Pezizomycotina	*Botryosphaeria dothidea*	*Botdot*	CBS 115476	JGI	130	4449	64	10740[a]	6390	10090	10452[a]
Pezizomycotina	*Botryotina fuckeliana* *(Botrytis cinerea)*	*Botfuc*	B05.10 + T4	SGD/NCBI	XP_001555197	XP_001548665.1	XP_001546494.1	XP_001549037.1	CCD44359.1	XP_001550869.1	CCD49853.1
Pezizomycotina	*Cercospora zeae-maydis*	*Cerzea*	SCOH1-5	JGI	115657	96381	43695	109721	65146	82003	40177
Pezizomycotina	*Chaetomium globosum*	*Chaglo*	CBS 148.51	BROAD/NCBI	XP_001230193	XP_001228221.1	XP_001220654	XP_001220925	CHGG_08507.1[a]	XP_001226555	XP_001225506
Pezizomycotina	*Cladonia grayi*	*Clagra*	Cgr/DA2myc/ss	JGI	22634	65732	117770	93669	26465	114585	113138 (a)
Pezizomycotina	*Claviceps fusiformis*	*Clafus*	PRL 1980	NCBI	Contig00388	AFRA01000298.1	AFRA01000003.1	AFRA01000069.1	AFRA01000949.1	AFRA01000186.1	AFRA01000143.1
Pezizomycotina	*Claviceps paspali*	*Clapas*	RRC 1481	NCBI	Contig00573	AFRC01000806.1	AFRC01000093.1	AFRC01000251.1	AFRC01000032.1	AFRC01000062.1	AFRC01000001.1
Pezizomycotina	*Coccidioides immitis*	*Cocimm*	RS	BROAD/NCBI	n.d.	XP_001243937.1	XP_001247351.1	XP_001243803.1	EAS29377.2	EAS30407.2	XP_001242390.1
Pezizomycotina	*Coccidioides posadasii*	*Cocpos*	C735 delta SOWgp	NCBI	n.d.	EFW20736.1	EFW21934.1	EFW20878.1	XP_003067891.1	EFW17285.1	XP_003069624.1
Pezizomycotina	*Cochliobolus heterostrophus*	*Cochet*	C5	JGI	1216790	93887	1191766	1217961	72858[a]	1186563	1206799

Phylum/ sub-phylum	Species	Abb.	Strain(s)	Genome database	Fsy1 (E < 1e-80)	Rpa1	Rpa2	Rpb1	Rpb2	Rpc1	Rpc2
Pezizomycotina	*Cochliobolus sativus*	*Cocsat*	ND90Pr	JGI	199593	189064	179601	152168	252499	195818	175739
Pezizomycotina	*Cochliobolus victoriae*	*Cocvic*	FI3	JGI	89654	91438	14336	111696	94880	85013	85595
Pezizomycotina	*Cordyceps militaris*	*Cormil*	CM01	NCBI	EGX91140	EGX92703.1	EGX88703.1	EGX91327.1	EGX90277.1	EGX94086.1	EGX93446.1
Pezizomycotina	*Cryphonectria parasitica*	*Crypar*	EP155	JGI	343078	88728	294188	81184	348358	47384	290714
Pezizomycotina	*Epichloe festucae*	*Epifes*	*Fl1*	NCBI	Contig00073	AFRX01000740.1	AFRX01000412.1	AFRX01000152.1	AFRX01000175.1	AFRX01000616.1	AFRX01000496.1
Pezizomycotina	*Epichloe typhina*	*Epityp*	*E5819*	NCBI	Contig00211	AFSE01000322.1	AFSE01000137.1	AFSE01000208.1	AFSE01000032.1	AFSE01000628.1	AFSE01000184.1
Pezizomycotina	*Eurotium herbariorum*	*Eurher*	CBS 516.65	JGI	452171	520297	386417	446535	412820	466346	379007
Pezizomycotina	*Exophiala dermatitidis*	*Exoder*	NIH/UT8656	BROAD	EHY58290.1	HMPREF1120_01885.1	HMPREF1120_06165.1	HMPREF1120_04534.1	HMPREF1120_04530.1	HMPREF1120_06710.1	HMPREF1120_02320.1
Pezizomycotina	*Fusarium graminearum* *(Gibberella zeae)*	*Fusgra*	PH-1	SGD/BROAD	XP_383344	XP_384467.1	XP_385803.1	XP_381092.1	FGSG_02659.3	XP_390036.1	XP_381019.1
Pezizomycotina	*Fusarium oxysporum*	*Fusoxy*	Fo5176	NCBI	EGU72941	EGU86828.1	EGU75766.1	EGU79617.1	EGU88188.1	EGU86224.1	EGU81124.1
Pezizomycotina	*Fusarium verticillioides* *(Gibberella moniliformis)*	*Fusver*	7600	SGD/BROAD	ABV60277	FVEG_11373.3	FVEG_06860.3	FVEG_00683.3	FVEG_09286.3	FVEG_02570.3	FVEG_00714.3
Pezizomycotina	*Gaeumannomyces graminis var. tritici*	*Gaegra*	R3-111a-1	BROAD/NCBI	n.d.	GGTG_10361.1	GGTG_01607.1	GGTG_01635.1	GGTG_09739.1	GGTG_09702.1	GGTG_10739.1
Pezizomycotina	*Geomyces destructans*	*Geodes*	20631-21	BROAD	GMDG_00621.1	GMDG_01895.1	GMDG_04700.1	GMDG_07930.1	GMDG_03987.1	GMDG_06549.1	GMDG_00002.1
Pezizomycotina	*Glarea lozoyensis*	*Glaloz*	74030	NCBI	EHK99759	EHK97632.1	EHK97205.1	EHK96688.1	EHL00094.1	EHL00082.1[a]	EHL03070.1
Pezizomycotina	*Glomerella graminicola* *(Colletotrichum graminicola)*	*Glogra*	M1.001	BROAD NCBI	CBS32704	GLRG_08740.1	EFQ29244.1	EFQ25440.1	EFQ28418.1	EFQ34067.1	EFQ33366.1
Pezizomycotina	*Grosmannia clavigera*	*Grocla*	kw1407	NCBI	EFX04626	EFX06682.1	EFX05208.1	EFW99454.1	EFX04627.1	EFX04724.1	EFX05916.1
Pezizomycotina	*Hysterium pulicare*	*Hyspul*	CBS 123377	JGI	n.d.	5865[a]	2240	4479	4193	10760	2101
Pezizomycotina	*Leptosphaeria maculans*	*Lepmac*	JN3	JGI	9120	CBY01975.1	Supercontig60_v2[a]	CBX97892.1[a]	CBX97713.1	CBY01243.1	CBX96804.1
Pezizomycotina	*Magnaporthe oryzae (grisea)*	*Magory*	70-15	BROAD/NCBI	XP_003714308	XP368086	XP363646	XP362207	XP362269	MGG_04477.6	XP370487
Pezizomycotina	*Magnaporthe poae*	*Magpoa*	73-15	BROAD/NCBI	n.d.	MAPG_02516.1	MAPG_06805.1	MAPG_06829.1	MAPG_02762.1	MAPG_02726.1	MAPG_00028.1
Pezizomycotina	*Metarhizium acridum*	*Metacr*	CQMa 102	NCBI	Contig598	ADNI01000893.1	ADNI01001037.1	ADNI01000981.1	ADNI01000325.1	ADNI01000058.1	ADNI01000743.1
Pezizomycotina	*Metarhizium anisopliae*	*Metani*	ARSEF 23	NCBI	EFZ02756	EFY96621.1	EFZ00762.1	EFZ03584.1	EFZ03262.1	EFY96310.1	EFZ02297.1
Pezizomycotina	*Myceliophthora thermophila*	*Mycthe*	ATCC 42464	JGI	n.d.	2312447	2295109	2294525	2307570	2307483	2312111
Pezizomycotina	*Mycosphaerella (Pseudocercospora) fijiensis*	*Mycfij*	CIRAD86	JGI	127211	64562	58897	58269	136855	187946	40745[a]
Pezizomycotina	*Mycosphaerella pini* *(Dothistroma septosporum)*	*Mycpin*	NZE10	JGI	119332	175298	155234	78752	42537	70566	125117
Pezizomycotina	*Mycosphaerella populicola* *(Septoria populicola)*	*Mycpou*	P02.02b	JGI	n.d.	32357	22730	17511	96839	22983	110855
Pezizomycotina	*Mycosphaerella populorum* *(Septoria musiva)*	*Mycpop*	SO2202	JGI	n.d.	86423	149523	62102	148144	147980	115106
Pezizomycotina	*Nectria haematococca*	*Nechae*	mpVI 77-13-4	JGI	XP_003047490	XP_003048471.1	XP_003048895.1	XP_003054466.1	XP_003045320.1	XP_003051073.1	XP_003054428.1
Pezizomycotina	*Neosartorya fischeri*	*Neofis*	NRRL 181	NCBI	XP_001257632	XP_001264366.1	XP_001257945.1	XP_001264289.1	XP_001262829.1	XP_001263030.1	XP_001265400.1
Pezizomycotina	*Neotyphodium gansuense*	*Neogan*	E7080	NCBI	Contig00506	AFRE01000286.1	AFRE01000467.1	AFRE01000377.1 AFRE01001118.1	AFRE01000108.1	AFRE01000118.1	AFRE01000230.1

**Phylum/ sub-phylum**	**Species**	**Abb.**	**Strain(s)**	**Genome database**	**Fsy1** (E < 1e-80)	**Rpa1**	**Rpa2**	**Rpb1**	**Rpb2**	**Rpc1**	**Rpc2**
Pezizomycotina	*Neurospora crassa*	*Neucra*	OR74A	BROAD/NCBI	n.d.	XP_956006.2	XP_964824.1	XP_964097.1	CAD70445.1	XP_956571.2	XP_957195.1
Pezizomycotina	*Neurospora discreta*	*Neudis*	FGSC 8579 mat A	JGI	n.d.	18787	94562	73118	128789	126699	18887
Pezizomycotina	*Neurospora tetrasperma*	*Neutet*	FGSC 2508 mat A	JGI	n.d.	EGO56440.1	EGO53799.1	EGO51638.1	EGO54804.1	EGO54506.1	EGO56562.1
Pezizomycotina	*Oidiodendron maius*	*Oidmai*	Zn	JGI	137033<br>177954<br>133091	198996	101491	129801	189344	92982	104825
Pezizomycotina	*Paracoccidioides brasiliensis*	*Parbra*	Pb01	BROAD/NCBI	n.d.	XP_002796309.1	EEH19405.1	XP_002792668.1	XP_002791308.1	XP_002794867.1	XP_002790004.1
Pezizomycotina	*Penicillium chrysogenum*	*Penchr*	Wisconsin 54-1255	NCBI	XP_002563779	XP_002561534.1	XP_002564802.1	XP_002561697.1	XP_002568295.1	XP_002559913.1	XP_002566073.1
Pezizomycotina	*Penicillium marneffei*	*Penmar*	ATCC 18224	NCBI	XP_002143393	XP_002146134.1	XP_002146749.1	XP_002146907.1	XP_002153141.1	XP_002149197.1	XP_002149081.1
Pezizomycotina	*Phaeosphaeria (stagonospora) nodorum*	*Phanod*	SN15	JGI	XP_001797741	XP_001805120.1	XP_001793675.1[a]	XP_001801796.1	ABF56194.1	XP_001792135.1	XP_001792395.1
Pezizomycotina	*Podospora anserina*	*Podans*	S mat+	NCBI/Genoscope	n.d.	XP_001910032.1	XP_001913030.1	XP_001912461.1	XP_001903788.1	XP_001903882.1	XP_001909583.1
Pezizomycotina	*Pyrenophora teres f. teres*	*Pyrter*	0-1	JGI	1306	XP_003296505.1	11055[a]	XP_003303389.1	XP_003299905.1	XP_003296505.1	XP_003304626.1
Pezizomycotina	*Pyrenophora tritici-repentis*	*Pyrtri*	Pt-1C-BFP	JGI	XP_001936502	XP_001932229.1	147694[a]	XP_001934953.1	XP_001934943.1	XP_001932816.1	XP_001933815.1
Pezizomycotina	*Rhytidhysteron rufulum*	*Rhyruf*	CBS 306.38	JGI	n.d.	7530	2990	1653	2106	5202	9460
Pezizomycotina	*Sclerotinia sclerotiorum*	*Sclscl*	1980 UF-70	BROAD/NCBI	XP_001596869	XP_001589882.1	XP_001585849.1	XP_001593006.1	XP_001598796.1	XP_001588093.1	XP_001595664.1
Pezizomycotina	*Setosphaeria turcica*	*Settur*	Et28A	JGI	24801	171714	179630	177979	177930	27903	183951
Pezizomycotina	*Sordaria macrospora*	*Sormac*	k-hell	NCBI	n.d.	CABT01000014.1	CABT01000040.1	CABT01000003.1	CABT01000044.1	CABT01000092.1	CABT01000014.1
Pezizomycotina	*Talaromyces stipitatus*	*Talsti*	ATCC 10500	NCBI	XP_002479713	XP_002478429.1	XP_002479042.1	XP_002479200.1	XP_002488312.1	XP_002484983.1	XP_002485094.1
Pezizomycotina	*Thielavia terrestris*	*Thiter*	NRRL 8126	JGI	2074954	XP_003657941.1	XP_003656793.1	XP_003656556.1	XP_003651004.1	XP_003650936.1	XP_003657789.1
Pezizomycotina	*Trichoderma atroviride*	*Triatr*	IMI 206040	JGI	284542	EHK48390.1	EHK47645.1	EHK40365.1	EHK44416.1	EHK42676.1	EHK46319.1
Pezizomycotina	*Trichoderma reesei*	*Triree*	QM6a	JGI	60945	EGR44276.1	EGR46237.1	EGR47538.1	EGR47390.1	EGR49933.1	EGR48063.1
Pezizomycotina	*Trichoderma virens*	*Trivir*	Gv29-8	JGI	86782	EHK17791.1	EHK17196.1	EHK25231.1	EHK23988.1	EHK22815.1	EHK15881.1
Pezizomycotina	*Trichophyton rubrum*	*Trirub*	CBS 118892	BROAD/NCBI	n.d.	XP_003231853.1	XP_003235733.1	XP_003231777.1	XP_003233873.1	XP_003232017.1	XP_003235084.1
Pezizomycotina	*Trichophyton tonsurans*	*Triton*	CBS112818	BROAD/NCBI	n.d.	EGD99288.1	EGD98439.1	EGD96855.1	EGD99045.1	EGD93894.1	EGD92650.1
Pezizomycotina	*Trichophyton verrucosum*	*Triver*	HKI 0517	BROAD/NCBI	n.d.	XP_003024908.1	XP_003021772.1	XP_003023110.1	XP_003025731.1	XP_003022324.1	XP_003020234.1
Pezizomycotina	*Uncinocarpus reeseii*	*Uncree*	1704	BROAD/NCBI	n.d.	XP_002582433.1	XP_002541550.1	XP_002582322.1	UREG_02143.1[a]	XP_002583147.1	XP_002544581.1
Pezizomycotina	*Verticillium dahliae*	*Verdah*	VdLs.17	BROAD/NCBI	n.d.	EGY20404.1	EGY21595.1	EGY15260.1	EGY14461.1	EGY15512.1	EGY15811.1
Pezizomycotina	*Zymoseptoria passarinii* (*Mycosphaerella passerinii*)	*Zympas*	SP63	NCBI	n.d.	AFIY01001176.1	AFIY01000078.1	AFIY01001095.1	AFIY01000537.1	AFIY01000789.1	AFIY01000642.1
Pezizomycotina	*Zymoseptoria tritici* (*Mycosphaerella graminicola*)	*Zymtri*	IPO323	JGI	n.d.	94196	73125	99873	99853	33399	36991[a]
Saccharomycotina	*Arxula adeninivorans*	*Arxade*		Génolevures & C. Neuvéglise (Acknowledgments)	ARAD1D05016g<br>ARAD1B17622g	ARAD1A03894g	ARAD1D42196g	ARAD1C32846g	ARAD1D00352g	ARAD1C14146g	ARAD1B19536g
Saccharomycotina	*Ascoidea rubescens*	*Ascrub*	NRRL Y17699	JGI	n.d.	158172	121398	152893	74872	37761	73423
Saccharomycotina	*Babjeviella inositovora*	*Babino*	NRRL Y-12698	JGI	n.d.	163242	159233	36081	159736	65579	160834

**Phylum/ sub-phylum**	**Species**	**Abb.**	**Strain(s)**	**Genome database**	**Fsy1** (E < 1e-80)	**Rpa1**	**Rpa2**	**Rpb1**	**Rpb2**	**Rpc1**	**Rpc2**
Saccharomycotina	*Candida tenuis*	*Canten*	NRRL Y-1498	JGI	n.d.	EGV63759.1	EGV64922.1	122800[a]	EGV66444.1	EGV61865.1	EGV61757.1
Saccharomycotina	*Candida albicans*	*Canalb*	WO-1	SGD/BROAD	CAWG_01680 CAWG_01693/01694	XP_710761.1	XP_720354.1	EEQ44066.1	XP_718439.1	EEQ44828.1	XP_710813.1
Saccharomycotina	*Candida arabinofermentans*	*Canara*	NRRL YB-2248	JGI	8968	237815	196656	28373	97166	5073	236521
Saccharomycotina	*Candida caseinolytica*	*Cancas*	NRRL Y-17796	JGI	n.d.	103917	71761	65867[a]	32658	57439[a]	76153
Saccharomycotina	*Candida dubliniensis*	*Candub*	CD36	SGD/YGOB	CD36_28610 CD36_28770	XP_002417643.1	XP_002421229.1	XP_002422523.1	XP_002416823.1	XP_002420293.1	XP_002422092.1
Saccharomycotina	*Candida glabrata*	*Cangla*	CBS 138	SGD/YGOB	n.d.	XP_445928.1	XP_447785.1	XP_447415.1	XP_448959.1	XP_449275.1	XP_448895.1
Saccharomycotina	*Candida parapsilosis*	*Canpar*	CDC317	BROAD/NCBI	CCE41657	CCE41808.1	CCE41117.1	CCE42368.1	CCE40898.1	CCE40988.1	CCE42811.1
Saccharomycotina	*Candida tanzawaensis*	*Cantan*	NRRL Y-17324	JGI	n.d.	27643	51620	205015	315886	20117	25811
Saccharomycotina	*Candida tropicalis*	*Cantro*	MYA-3404	SGD/BROAD	CTRG_00752 CTRG_00741	XP_002548986.1	XP_002550740.1	Supercontig10[d]	XP_002550158.1	XP_002546294.1[d]	XP_002545804.1
Saccharomycotina	*Candida zemplinina*	*Canzem*	PYCC 3044	GenBank (local genome database)	n.d.	KF055412 (scaffold00024)	KF055413 (scaffold00010)	KF055414 (scaffold00035)	KF055415 (scaffold00058)	KF055416 (scaffold00059)	KF055417 (scaffold00001)
Saccharomycotina	*Clavispora lusitaniae*	*Clalus*	ATCC 42720	BROAD/NCBI	XP_002616715[c]	XP_002614662.1	XP_002617668.1	XP_002616227.1	CLUG_03629.1[a]	XP_002617549.1	XP_002617014.1
Saccharomycotina	*Cyberlindnera jadinii*	*Cybjad*	NBRC 0988	NCBI	Chr4_Contig9 (pseudogene)	BAEL01000045.1	BAEL01000080.1	BAEL01000145.1	BAEL01000002.1[d]	BAEL01000017.1	BAEL01000068.1
Saccharomycotina	*Debaryomyces hansenii*	*Debhan*	CBS767	SGD/NCBI	CAR66377	XP_002770194.1	XP_461338.1	XP_002770038.1	XP_002770594.1	XP_462102.2	XP_002777730.1
Saccharomycotina	*Dekkera bruxellensis*	*Dekbru*	AWRI1499	NCBI	n.d.	68261	EIF46931.1	EIF46824.1	7243	EIF47620.1	87139
Saccharomycotina	*Eremothecium cymbalariae*	*Erecym*	DBVPG#7215	NCBI/YGOB	n.d.	Chr 5	XP_003644970.1	XP_003648178.1	XP_003646391.1	XP_003647083.1	XP_003648395.1
Saccharomycotina	*Eremothecium gossypii*	*Eregos*	ATCC 10895	SGD/NCBI	n.d.	NP_984470.2	NP_982975.2	NP_984182.2	NP_985951.1	NP_985109.2	NP_983821.2
Saccharomycotina	*Hyphopichia burtonii*	*Hypbur*	NRRL Y-1933	JGI	107034	235620	10853	213139	152096	203469	105789
Saccharomycotina	*Kazachstania africana*	*Kazafr*	CBS 2517	YGOB	n.d.	CCF60409.1	CCF57324.1	CCF56404.1	CCF58398.1	CCF58150.1	CCF59234.1
Saccharomycotina	*Kazachstania naganishii*	*Kaznag*	CBS 8797	YGOB	n.d.	Chr 4	Chr 4	Chr 3	Chr 2	Chr 2	Chr 1
Saccharomycotina	*Kluyveromyces aestuarii*	*Kluaes*	ATCC 18862	NCBI	AEAS01000322.1	AEAS01000160.1	AEAS01000155.1	AEAS01000088.1	AEAS01000100.1	AEAS01000212.1	AEAS01000006.1
Saccharomycotina	*Kluyveromyces lactis*	*Klulac*	NRRL Y-1140	SGD/YGOB	XP_454356	XP_454356	XP_456115.1	XP_451816.1	XP_455310.1	XP_451784.1	XP_454912.1
Saccharomycotina	*Kluyveromyces wickerhamii*	*Kluwic*	UCD 54-210	NCBI	AEAV01000496.1	AEAV01000083.1	AEAV01000033.1	AEAV01000150.1	AEAV01000240.1	AEAV01000216.1	AEAV01000246.1
Saccharomycotina	*Komagataella pastoris*	*Kompas*	GS115 + CBS 7435	SGD/NCBI	n.d.	XP_002491799.1	XP_002490446.1	XP_002492795.1	XP_002491011.1	CCA40852.1	XP_002493647.1
Saccharomycotina	*Lachancea kluyveri*	*Lacklu*	NRRL Y-12651	SGD/YGOB	SAKL0E06864g	Chr 2	Chr 2	Chr 6	Chr 7	Chr 7	Chr 8
Saccharomycotina	*Lachancea thermotolerans*	*Lacthe*	CBS 6340	SGD/YGOB	XP_002555518	XP_002552795.1	XP_002555777.1	XP_002555968.1	XP_002555168.1	XP_002554891.1	XP_002553136.1
Saccharomycotina	*Lachancea waltii*	*Lacwal*	NCYC 2644	SGD/YGOB	Kwal_27.11385[c]	kwal_s_26	kwal_s_14	kwal_s_56	kwal_s_47	kwal_s_55	kwal_s_26
Saccharomycotina	*Lipomyces starkeyi*	*Lipsta*	NRRL Y-11557	JGI	109777	336068	4553	75854	73345	1849	331
Saccharomycotina	*Lodderomyces elongisporus*	*Lodelo*	NRRL YB-4239	BROAD/NCBI	XP_001527752	XP_001527964.1	XP_001523265.1	XP_001523387.1	XP_001526602.1	XP_001523103.1	XP_001525350.1
Saccharomycotina	*Metschnikowia bicuspidata*	*Metbic*	NRRL YB-4993	JGI	76577	101699	30063	170566	32161	12577	32049
Saccharomycotina	*Meyerozyma guilliermondii*	*Meygui*	ATCC 6260	BROAD/NCBI	XP_001486924	EDK39611.2	EDK36311.2[a,d]	XP_001487729.1	EDK38735.2	EDK40245.2	EDK39998.2
Saccharomycotina	*Millerozyma farinosa*	*Milfar*	CBS 7064	SGD/NCBI	CCE87359	CCE81062.1	CCE86373.1	CCE87208.1	CCE78311.1	CCE88859.1	CCE82943.1

Phylum/ sub-phylum	Species	Abb.	Strain(s)	Genome database	Fsy1 (*E* < 1e-80)	Rpa1	Rpa2	Rpb1	Rpb2	Rpc1	Rpc2
Saccharomycotina	*Nadsonia fulvescens var. elongata*	*Nadflu*	DSM 6958	JGI	n.d.	49068	50775	44819	82421	70621	53794
Saccharomycotina	*Naumovozyma castellii*	*Naucas*	CBS 4309	YGOB	n.d.	XP_003676913.1	XP_003675778.1	XP_003674328.1	XP_003677897.1	XP_003677861.1	XP_003674628.1
Saccharomycotina	*Naumovozyma dairenensis*	*Naudai*	CBS 421	YGOB	n.d.	XP_003671537.1	XP_003671155.1	XP_003667614.1	XP_003669029.1	XP_003669066.1	XP_003670214.1
Saccharomycotina	*Ogataea angusta*	*Ogaang*	NCYC 495 leu1.1	JGI	15869	64765	28517	62103	64873	17249[a]	64464
Saccharomycotina	*Ogataea parapolymorpha*	*Ogapar*	DL-1	NCBI	EFW97034	EFW96323.1	EFW95149.1	EFW94818.1	EFW96371.1	EFW95798.1	EFW95940.1
Saccharomycotina	*Pachysolen tannophilus*	*Pactan*	NRRL Y-2460	JGI/NCBI	n.d.	CAHV01000103.1	CAHV01000123.1	CAHV01000062.1	CAHV01000236.1[d]	CAHV01000062.1	CAHV01000160.1
Saccharomycotina	*Pichia kudriavzevii*	*Pickud*	M12	NCBI	n.d.	ALNQ01000183.1	ALNQ01000058.1	ALNQ01000167.1	ALNQ01000064.1	ALNQ01000075.1	ALNQ01000054.1
Saccharomycotina	*Pichia membranifaciens*	*Picmem*	NRRL Y-2026	JGI	n.d.	15631	15732	28620	71110	73178	15919
Saccharomycotina	*Saccharomyces cerevisiae*	*Saccer*	S288c	SGD/BROAD	n.d.	P10964.2	P22138.1	P04050.2	P08518.2	P04051.1	P22276.2
Saccharomycotina	*Saccharomyces cerevisiae*	*Saccer*	EC1118	NCBI	CAY86682						
Saccharomycotina	*Saccharomyces eubayanus*	*Saceub*	FM1318	[Ref. 29]	CCI61473	Contig0673	Contig0661	Contig0455 Contig0592[b]	Contig587	Contig620	Contig583[d]
Saccharomycotina	*Saccharomyces kudriavzevii*	*Sackud*	IFO 1802	SSS Website	n.d.	Scaffold8	Scaffold34	Scaffold3	Scaffold58	Scaffold51	Scaffold8
Saccharomycotina	*Saccharomyces mikatae*	*Sacmik*	IFO 1815	SSS Website SGD/BROAD	n.d.	Scaffold13	Scaffold1	Scaffold8	Scaffold32	Scaffold32	Scaffold13
Saccharomycotina	*Saccharomyces paradoxus*	*Sacpar*	NRRLY-17217	SSS Website NCBI	n.d.	Scaffold15	Scaffold16	AABY01000121.1[d]	Scaffold15	AABY01000093.1[d]	Scaffold15
Saccharomycotina	*Saccharomyces uvarum*	*Sacuva*	CBS 7001	SSS Website SGD/BROAD	AEO27991	Scaffold8	Scaffold2	Scaffold11	Scaffold8	Scaffold16	Scaffold8
Saccharomycotina	*Scheffersomyces stipitis*	*Schsti*	CBS 6054	JGI/NCBI	XP_001385662	XP_001386054.2	XP_001384965.2	XP_001386996.2	XP_001387366.2	XP_001387687.2	XP_001383024.1
Saccharomycotina	*Spathaspora passalidarum*	*Spapas*	NRRL Y-27907	JGI/NCBI	EGW34319	EGW32330.1	EGW30093.1	EGW33664.1	EGW34678.1	EGW35786.1	EGW34142.1
Saccharomycotina	*Tetrapisispora blattae*	*Tetbla*	CBS 6284	YGOB	n.d.	CCH61247.1	CCH61446.1	CCH61759.1	CCH58045.1	CCH58170.1	CCH59253.1
Saccharomycotina	*Tetrapisispora phaffii*	*Tetpha*	CBS 4417	YGOB	n.d.	XP_003686917.1	XP_003686698.1	XP_003683819.1	XP_003687546.1	XP_003686816.1	XP_003683621.1
Saccharomycotina	*Torulaspora delbrueckii*	*Tordel*	CBS 1146	YGOB	XP_003683518	XP_003683470.1	XP_003679887.1	XP_003680332.1	XP_003678904.1	XP_003681653.1	XP_003679108.1
Saccharomycotina	*Vanderwaltozyma polyspora*	*Vanpol*	DSM 70294	YGOB/NCBI	n.d.	XP_001643648.1	XP_001645652.1	XP_001642173.1	XP_001642238.1	XP_001643927.1	XP_001643385.1
Saccharomycotina	*Wickerhamomyces anomalus*	*Wicano*	NRRL Y-366	JGI	103220	62320	99340	60320	53842	31590	36757[a]
Saccharomycotina	*Yarrowia* (*Candida*) *hispaniensis*	*Yarhis*		C. Neuvéglise (Acknowledgments)	n.d.	scf4345 [2562413-2557724]	scf4343 [798972-802445]	scf4340 [807063-802168]	scf4345 [1779245-1782919]	scf4345 [1469871-1474277]	scf4341 [201460-204927]
Saccharomycotina	*Yarrowia lipolytica*	*Yarlip*	CLIB122	SGD/NCBI	n.d.	XP_505388.1	XP_503752.1	XP_501909.2	XP_502376.1	XP_502142.1	XP_500966.1
Saccharomycotina	*Zygosaccharomyces rouxii*	*Zygrou*	CBS 732	SGD/YGOB	XP_002495678	XP_002496630.1	XP_002499065.1	XP_002497454.1	XP_002496945.1	XP_002497625.1	XP_002497679.1
Taphrinomycotina	*Saitoella complicata*	*Saicom*	NRRL Y-17804	JGI	n.d.	54019	10334	88126[a]	33216[a]	54128	65571
Taphrinomycotina	*Schizosaccharomyces japonicus*	*Schjap*	yFS275	BROAD/NCBI	n.d.	XP_002174310.1	XP_002174716.1	XP_002172959.1	XP_002175152.1	XP_002176004.1	XP_002171911.1
Taphrinomycotina	*Schizosaccharomyces octosporus*	*Schoct*	yFS286	BROAD	n.d.	SOCG_00187.1	SOCG_01988.1	SOCG_00001.1	SOCG_02073.1	SOCG_03410.1	SOCG_04311.1
Taphrinomycotina	*Schizosaccharomyces pombe*	*Schpom*	972h-	NCBI	n.d.	NP_596300.1	NP_595819.2	NP_595673.1	NP_593101.2	NP_595506.1	NP_593690.1
