# Supplementary material for: Extensive Intra-Kingdom Horizontal Gene Transfer Converging on a Fungal Fructose Transporter Gene
Source: PLoS Genet. 2013 Jun 20;9(6):e1003587. doi: 10.1371/journal.pgen.1003587 (PMC3688497; doi:10.1371/journal.pgen.1003587)
Supplement: Table S2 — Constraint analysis for each of the presumed HGT events involving Fsy1. The Shimodaira-Hasegawa (SH) test as implemented in RAxML was used to compare Fsy1 constrained topologies with the Fsy1 ML topology. P values were considered significant if less than 0.05. (PDF) [file pgen.1003587.s009.pdf]

Table S2 – Constraint analysis for each of the presumed HGT events involving Fsy1.

HGT event(s)	Likelihood of the optimal tree	Likelihood of the constrained tree	D (LH)	SD	Significantly worse?	*P*-value
**1**		-19955.513335	-98.512891	24.598493	Yes	$P < 0.01$
**2**		-20050.539689	-193.539245	26.222165	Yes	$P < 0.01$
**3a**		-20045.620987	-188.620543	27.329450	Yes	$P < 0.01$
**3b**		-19982.676517	-125.676073	24.705889	Yes	$P < 0.01$
**2 & 3b**		-20157.436066	-300.435622	42.474248	Yes	$P < 0.01$
**4**		-19931.353591	-74.353148	17.485860	Yes	$P < 0.01$
**5**		-19985.758468	-128.758024	24.146184	Yes	$P < 0.01$
**6**		-20045.381908	-188.381464	26.437025	Yes	$P < 0.01$
**7**		-20769.632249	-912.631805	69.435386	Yes	$P < 0.01$
**8, 9 & 10**	-19857.000444	-19862.384812	-5.384368	6.857076	No	
**11a****		-19873.364170	-16.363726	8.744431	No	
**11b*****		-19867.252462	-10.252018	6.096686	No	
**12**		-19880.359227	-23.358783	10.226561	Yes	$P < 0.05$
**13**		-19884.307385	-27.306941	12.291208	Yes	$P < 0.05$
**14***		*				
**Pezizomycotina to Saccharomycotina***		*				

*No constraint was possible as Fsy1 and species tree topologies are congruent for these branches.
**Orthologues from CUG clade species were forced to form a monophyletic group.
***Orthologues from CUG clade species, except Milfar1 and Milfar2, were forced to form a monophyletic group. Milfar1 and Milfar2 maintained their phylogenetic position as depicted in the Fsy1 tree.
