# Supplementary material for: Extensive Intra-Kingdom Horizontal Gene Transfer Converging on a Fungal Fructose Transporter Gene
Source: PLoS Genet. 2013 Jun 20;9(6):e1003587. doi: 10.1371/journal.pgen.1003587 (PMC3688497; doi:10.1371/journal.pgen.1003587)
Supplement: Table S4 — List of primers, specific PCR conditions, plasmid constructs and strains used for heterologous expression of FSY1 homologues in S. cerevisiae hxt-null. Primer sequences and annealing temperatures used to amplify each FSY1 homologue are indicated. Plasmids containing FSY1 homologues derived from the indicated fungal species were constructed by homologous recombination in S. cerevisiae EBY.VW4000. aprimer used for first-strand cDNA synthesis. bprimers with overhangs identical to the 3′ end of TEF promoter (underlined) and the 5′ end of the CYC1 terminator (bold) used for homologous recombination into the p415 TEF plasmid. T Type strain. (PDF) [file pgen.1003587.s011.pdf]

Table S4 – List of primers, specific PCR conditions, plasmid constructs and strains used for heterologous expression of *FSY1* homologues in *S. cerevisiae hxt*-null.

**Species/strain**	**Gene**	**Primers**	**Primer Sequence (5' → 3')**	**Annealing temperature**	**Plasmids**
A. niger (ATCC 16404)	*FSY1*	An_p415FSY_Fw[b]	**GAAAGAAAGCATAGCAATCTAATCTAAGTTTTAATTACAAATC**ATGGGTGTCGTACACGACCTG	59ºC	pCGFRUY24
		An_p415FSY_Rv[b]	TGAATGTAAGCGTGACATAACTAATTACATGACTCGTCAGTCCTCCTTCGGCGGC		
		An_FSY_Fw	ATGGGTGTCGTACACGACCTG	59ºC	
		An_FSY_Rv[a]	TCAGTCCTCCTTCGGCGGCG		
	"yeast-like" *FSY1*	An_p415FSYb_Fw[b]	**GAAAGAAAGCATAGCAATCTAATCTAAGTTTTAATTACAAATC**ATGGGCGATAATTATGAGGA	52ºC	pCGFRUY19
		An_p415FSYb_Rv[b]	TGAATGTAAGCGTGACATAACTAATTACATGACTCGTTAGGCGTTGTAGTTATC		
		An_FSYb_Fw	ATGGGCGATAATTATGAGGA	52ºC	
		An_FSYb_Rv	TTAGGCGTTGTAGTTATC		
C. albicans (PYCC 3436[T])	*FSY1*	Calb_p415FSY_Fw[b]	**GCATAGCAATCTAATCTAAGTTTTAATTACAAATCTAGA**ATGCTGTCTGTTTCACTGGAAAAG	56ºC	pCGFRUY22
		Calb_p415FSY_Rv[b]	CGTGAATGTAAGCGTGACATAACTAATTACATGACTCGAGTCAACGGCCCCAGAACCTC		
		Calb_FSY_Fw	ATGCTGTCTGTTTCACTGGA	56ºC	
		Calb_FSY_Rv[a]	TCAACGGCCCCAGAACCTCTT		
C. arabinofermentans (PYCC 5603[T])	*FSY1*	Cara_p415FSY_Fw[b]	**GCATAGCAATCTAATCTAAGTTTTAATTACAAATCTAGA**ATGTCTAATATTAATGAAGT	46ºC	pCGFRUY17
		Cara_p415FSY_Rv[b]	CGTGAATGTAAGCGTGACATAACTAATTACATGACTCGAGGTAAAAATGTTGGTTATTAG		
L. starkeyi (PYCC 4045[T])	*FSY1*	Lstar_p415FSY_Fw[b]	**GCATAGCAATCTAATCTAAGTTTTAATTACAAATCTAGA**ATGGTTTCTGAGAAGAAATTGC	54ºC	pCGFRUY21
		Lstar_p415FSY_Rv[b]	CGTGAATGTAAGCGTGACATAACTAATTACATGACTCGAGCTAAGCCTCTCCCTCCC		
		Lstar_FSY_Fw	ATGGTTTCTGAGAAGAAATTGCA	54ºC	
		Lstar_FSY_Rv	CTAAGCCTCTCCCTCCC		
S. uvarum (CBS 7001)	*FSY1*	Suva_p415FSY_Fw[b]	**GAAAGAAAGCATAGCAATCTAATCTAAGTTTTAATTACAAATC**ATGTCTCATGTTAACGCGTC	52ºC	pCGFRUY26
		Suva_p415FSY_Rv[b]	TGAATGTAAGCGTGACATAACTAATTACATGACTCGTTAATAACTCAATTGGCCCTT		
		Suva _FSY_Fw	ATGTCTCATGTTAACGCGTC	52ºC	
		Suva_FSY_Rv	TTAATAACTCAATTGGCCCTT		

S. cerevisiae (EBY.VW4000)	*MATα leu2-3,112 ura3-52 trp1-289 his3-Δ1 MAL2-8*[c] *SUC2 hxt17Δ hxt13Δ::loxP hxt15Δ::loxP hxt16Δ::loxP hxt14Δ::loxP hxt12Δ::loxP hxt9Δ::loxP hxt11Δ::loxP hxt10Δ::loxP hxt8Δ::loxP hxt514Δ::loxP hxt2Δ::loxP hxt367Δ::loxP gal2Δ snf3Δ::loxP stl1Δ::loxP agt1Δ::loxP ydl247wΔ::loxP yjr160cΔ::loxP*	Ref. [50]
